# A cancer-associated RNA polymerase III identity drives robust transcription and expression of snaR-A noncoding RNA

**DOI:** 10.1038/s41467-022-30323-6

**Published:** 2022-05-30

**Authors:** Kevin Van Bortle, David P. Marciano, Qing Liu, Tristan Chou, Andrew M. Lipchik, Sanjay Gollapudi, Benjamin S. Geller, Emma Monte, Rohinton T. Kamakaka, Michael P. Snyder

**Affiliations:** 1 Department of Genetics, Stanford University, Stanford, CA 94305 USA; 2 Department of Cell & Developmental Biology, University of Illinois Urbana-Champaign, Urbana, IL 61801 USA; 3 Cancer Center at Illinois, University of Illinois Urbana-Champaign, Urbana, IL 61801 USA; 4 Stanford Cardiovascular Institute, Stanford University School of Medicine, Stanford, CA 94305 USA; 5 Department of Physiology, Southern Illinois University School of Medicine, Carbondale, IL 62901 USA; 6 Department of Pharmaceutical Sciences, Eugene Applebaum College of Pharmacy and Health Sciences, Wayne State University, Detroit, MI 48201 USA; 7 Genomics Research Internship Program at Stanford, Stanford University, Stanford, CA 94305 USA; 8 Department of Molecular, Cell, and Developmental Biology, University of Santa Cruz, Santa Cruz, CA 95064 USA

**Keywords:** Transcription, Small RNAs, Cancer genomics, Long non-coding RNAs, Cell proliferation

## Abstract

RNA polymerase III (Pol III) includes two alternate isoforms, defined by mutually exclusive incorporation of subunit POLR3G (RPC7α) or POLR3GL (RPC7β), in mammals. The contributions of POLR3G and POLR3GL to transcription potential has remained poorly defined. Here, we discover that loss of subunit POLR3G is accompanied by a restricted repertoire of genes transcribed by Pol III. Particularly sensitive is snaR-A, a small noncoding RNA implicated in cancer proliferation and metastasis. Analysis of Pol III isoform biases and downstream chromatin features identifies loss of POLR3G and snaR-A during differentiation, and conversely, re-establishment of *POLR3G* gene expression and *SNAR-A* gene features in cancer contexts. Our results support a model in which Pol III identity functions as an important transcriptional regulatory mechanism. Upregulation of *POLR3G*, which is driven by MYC, identifies a subgroup of patients with unfavorable survival outcomes in specific cancers, further implicating the POLR3G-enhanced transcription repertoire as a potential disease factor.

## Abstract (web summary)

RNA polymerase III changes its subunit composition during mammalian development. Here the authors report that loss of subunit POLR3G, which re-emerges in cancer, promotes expression of small NF90-associated RNA (snaR-A), a noncoding RNA implicated in cell proliferation and metastasis.

## Introduction

The cellular division of transcriptional labor includes the RNA polymerase III (Pol III) apparatus, specially tuned for production of small, noncoding RNAs critical for translation, transcriptional and post-transcriptional regulation, and other fundamental processes^1^. Structural and biochemical studies of the Pol III complex, gene promoter architectures, and essential transcriptional regulators have extensively decoded the Pol III transcriptome and layers of transcriptional regulation^2–5^. Well-defined classes of small noncoding RNA (ncRNA) synthesized by Pol III include tRNA and 5 S ribosomal RNA (translation), 7SK and U6 small nuclear RNA (Pol II regulation and pre-mRNA splicing, respectively), RMRP and H1 (processing of rRNA and tRNA, respectively), and 7SL (RNA scaffold of the signal recognition particle)^6–15^. Additional classes of Pol III-transcribed ncRNA, whose functions remain less well understood, include vault RNA (regulation of apoptosis and autophagy)^16,17^, Y RNA (replication initiation; translation regulation)^18,19^, BC200 (translation regulation)^20^, and snaR RNA, which interacts with RNA-binding protein ILF3^21,22^. BC200 and snaR-A, a specific isoform of snaR ncRNA, are upregulated in a variety of cancer contexts, yet the underlying mechanisms and disease contributions of these ncRNA species remain poorly understood^23–27^.

Three distinct promoter architectures categorically classify Pol III-transcribed genes based on internal (type I, II) and external (type III) DNA sequence features and complementary transcription factor repertoires^28^. Pol III recruitment and activity is also controlled through interactions with oncogenic and tumor-suppressor signaling pathways, connecting growth-related synthesis of tRNA and other small RNAs to extracellular growth cues^29^. Specific pathways converge on the transcription factor complex, TFIIIB, as well as MAF1, a transcriptional repressor that antagonizes Pol III occupancy and activity^30–34^. However, MAF1 is a chronic repressor of Pol III activity in both proliferative and differentiated contexts, suggesting additional mechanisms may also contribute to context-specific Pol III transcription regulation at the gene level^35^. Previous genomic mapping studies of Pol III occupancy suggest some level of tissue-specific activity patterns^36–39^, and we and others have reported dynamic loss of Pol III occupancy and transcription at specific tRNA genes during cellular differentiation and exit from proliferation^40,41^. Comprehensive profiles of Pol III activity within cellular models of development are needed to better understand the level of dynamic transcription at other classes of Pol III-transcribed genes, and the mechanisms contributing to restricted Pol III activity during differentiation.

Structurally, Pol III is the largest RNA polymerase protein complex and is comprised of multiple subunits that are either Pol III-specific or otherwise shared with Pol I and/or Pol II^42^. Unique components include the large subunits, POLR3A (RPC1) and POLR3B (RPC2), which constitute the catalytic DNA-binding core, POLR3D (RPC4) and POLR3E (RPC5), which establish a stably associated subcomplex involved in transcription initiation and termination, as well as POLR3C (RPC3), POLR3F (RPC6), and POLR3G (RPC7α), which together form a heterotrimeric subcomplex required for recruitment of Pol III via direct interactions with transcription factor complex, TFIIIB^43–47^. POLR3G is highly expressed during early development and subsequently attenuated during differentiation^48,49^. A paralogous subunit, POLR3GL (RPC7β), is expressed at later stages of development and required for long-term survival in vivo^50^. Replacement of POLR3G and incorporation of POLR3GL into the analogous RPC3-RPC6-RPC7 heterotrimeric subcomplex during development imply that Pol III-transcribed genes are expressed by distinct complex identities depending on the spatiotemporal regulation of *POLR3G* and *POLR3GL* genes^51^.

Subunits POLR3G and POLR3GL share 46% amino acid identity and were established by a gene duplication event in the common ancestor of vertebrates^52^. Whether differences in Pol III composition defined by POLR3G or POLR3GL mechanistically drive unique transcription patterns has not been well established. However, initial mapping of POLR3G and POLR3GL, as well as functional analysis of these subunits in mice, suggests a strong level of redundancy with respect to gene occupancy and contribution to developmental potential, respectively^50,52^. Despite evidence of redundancy, ectopic expression of *POLR3G*, but not *POLR3GL*, enhances RNA levels for a subset of Pol III-transcribed genes in IMR90 cells, and knockdown of *POLR3G* in human pluripotent stem cells induces changes in specific Pol III-transcribed ncRNA levels^49,51^. Recent Pol III structural analysis suggests that interactions between the conserved C-terminal tail regions of POLR3G and POLR3GL and the active center of Pol III may function as an autoinhibitory mechanism that precludes transcription in the absence of direct interactions with TFIIIB^53^. While potential differences in POLR3G- and POLR3GL-mediated autoinhibition require further investigation, additional structural insight suggests that subunit POLR3G, but not POLR3GL, may block the site of MAF1 interaction required for repression of Pol III activity^54^. These findings imply that POLR3G and POLR3GL may not be strictly redundant, and that complex identity may indeed exert some level of context-specific control of Pol III transcription potential.

**Fig. 1:**
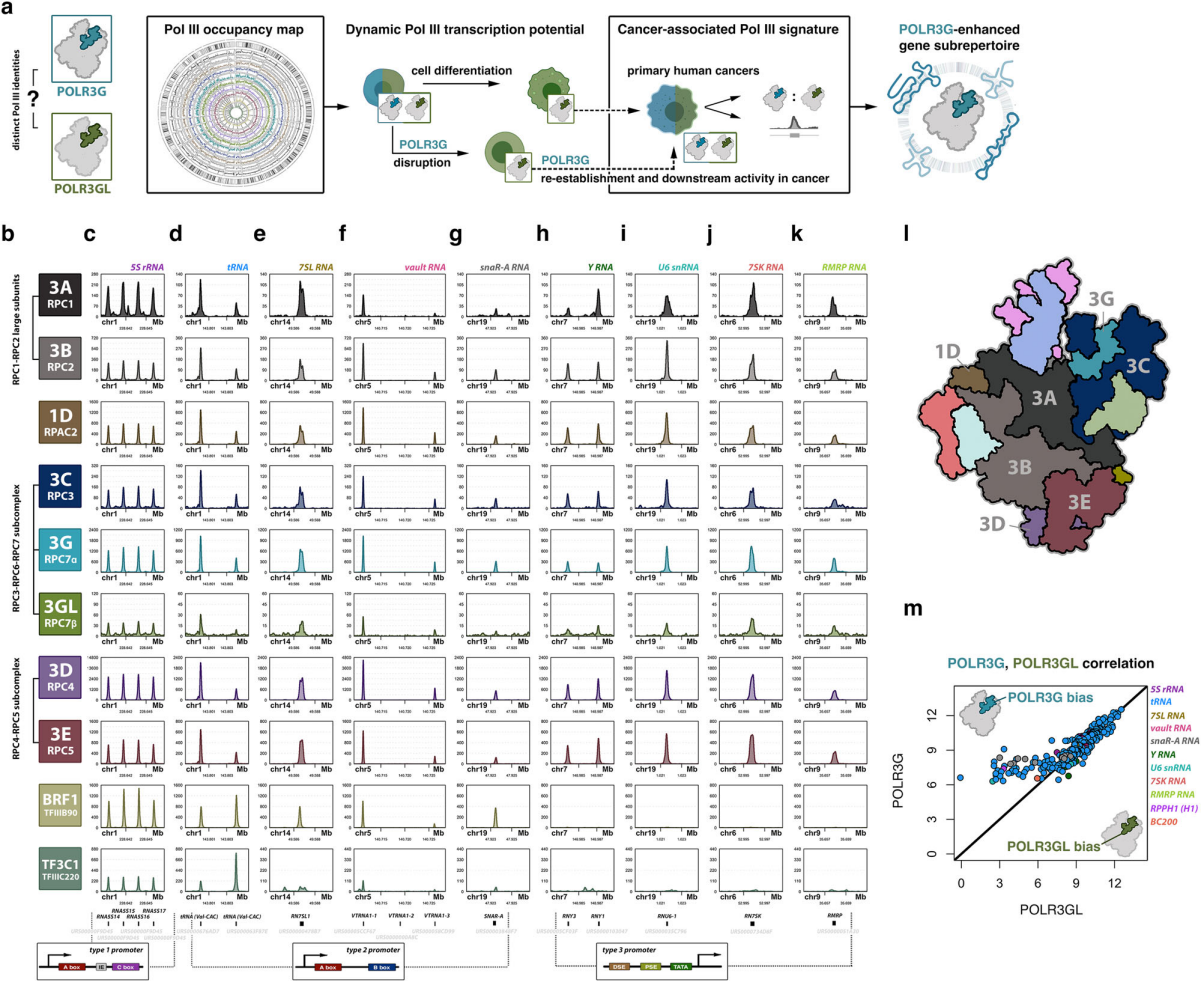
Study workflow and combinatorial atlas of human RNA Polymerase III subunits across canonical Pol III-transcribed genes. **a** Illustration of experimental approach tracing the effect of developmental loss of subunit POLR3G, subunit-specific disruption of POLR3G, and cancer-associated re-establishment of POLR3G and accompanying chromatin features, which collectively identify POLR3G-driven modulation of Pol III transcription potential in proliferating cells. **b** Pol III subunit and transcription factor legend corresponding to genome-wide maps in panels **c**–**k**, including POLR3A/RPC1 (3A); POLR3B/RPC2 (3B); POLR1D/RPAC2 (1D); POLR3C/RPC3 (3C); POLR3G/RPC7α (3G); POLR3GL/RPC7β (3GL); POLR3D/RPC4 (3D); POLR3E/RPC5 (3E); BRF1/TFIIIB90; TF3C1/TFIIIC220. Corresponding subcomplex indicated. **c**–**k** Example ChIP-seq read signals for each subunit/factor are shown in THP-1 monocytes across canonical Pol III-transcribed genes of varying promoter architecture, including ribosomal 5 S rRNA genes (type 1 promoter; panel **c**), tRNA, 7SL, vault, and snaR-A ncRNA genes (type 2 promoter, panels **d**–**g**, respectively), and Y, U6, 7SK, and RMRP ncRNA genes (type 3 promoter, panels **h**–**k**, respectively). Gene labels include Unique RNA Sequence (URS) identifiers assigned by (and connected to) the RNAcentral database. Bottom panel illustrates corresponding promoter architecture classification. **l** Cartoon schematic of the Pol III protein complex with emphasis on subunits mapped in this study (labeled) and the corresponding color code for genomic signal plots. Illustration serves as general reference guide inspired by previous structural reconstructions (Hoffmann et al., Nature 2015)^46^. **m** Visualization of the correlation between log2(normalized read densities) for paralogous Pol III subunits, POLR3G and POLR3GL, at Pol III complex–occupied genes in THP-1 monocytes. Source Data are provided as a Source Data file.

To better understand the role of subunit composition as a potential underlying mechanism of Pol III transcription regulation, we established an unprecedented genomic map of Pol III subunit occupancy and transcription in proliferating THP-1 monocytes and traced the effects of POLR3G loss on Pol III activity during differentiation and experimental disruption (Fig. 1a). We additionally investigated the levels of POLR3G and POLR3GL expression and downstream chromatin features in large-scale analyses of primary immune cells and primary solid tumors. These experiments uncover a strong relationship between POLR3G availability and competent Pol III transcription of *SNAR-A* gene clusters, specifically, and expansion of Pol III transcription potential, generally. Our results suggest developmental loss of POLR3G restricts the transcription potential of Pol III, supporting a nonredundant model in which subunits POLR3G and POLR3GL contribute to a dynamic transcriptional landscape.

## Results

### A combinatorial atlas of Pol III occupancy across canonical Pol III–transcribed genes

We used a combinatorial genomic approach to identify active Pol III–transcribed genes, relying on the extended fragment size and corresponding sequence reads of chromatin immunoprecipitation (ChIP) experiments to confidently identify Pol III complex localization. Measures of Pol III occupancy are subsequently integrated with profiles of nascent and steady–state small RNA abundance. Included in our expansive genome–wide Pol III map are the binding profiles for both large subunits, POLR3A and POLR3B, shared subunit POLR1D, multiple subunits of the RPC3-RPC6-RPC7 heterotrimer subcomplex involved in transcription initiation, including POLR3C, POLR3G, and POLR3GL, and both subunits of the RPC4-RPC5 heterodimer subcomplex involved in transcription initiation and termination, POLR3D and POLR3E (Fig. 1b–l). We additionally profiled the binding patterns of BRF1, a subunit of general transcription factor TFIIIB, as well as TF3C1, the largest subunit of TFIIIC (Fig. 1b–k). The overlap of all mapped Pol III subunits at specific genomic elements is a confident indicator of Pol III transcription and, in this respect, is a valuable resource for identifying the gene repertoire of Pol III activity within this system. Pol III and transcription factor ChIP signals were analyzed over a comprehensive list of canonical Pol III-transcribed gene coordinates using RNAcentral (https://rnacentral.org), a collection of multiple noncoding RNA annotation databases^55,56^.

In total, we identified 350 canonical Pol III-transcribed genes enriched for concordant Pol III ChIP signal in our cell culture system (Supplementary Fig. 1a). Inspection of individual subunit ChIP-seq signal intensities confirm occupancy across all classes of Pol III-transcribed genes, including genes encoding 5 S rRNA, tRNA, 7SL, vault, snaR, Y, U6, 7SK, and RMRP RNA (Fig. 1c–k). BRF1 and TF3C1 occupancies are restricted to type 1 and type 2 promoter architectures but absent at type 3 gene promoters, consistent with the established mechanisms and transcription factor repertoires underlying Pol III transcription initiation across genes with distinct internal and upstream promoter sequence features^28^. Overall, maps of Pol III subunit occupancy are broadly consistent across individual Pol III subunits, with specific genes featuring high levels of signal intensity across each subunit, and other genes featuring moderate to low signal intensity for all mapped subunits. Rank normalization of Pol III and transcription factor signal intensities illustrate this relationship, with high occupancy genes featuring strong signals for all subunits (Supplementary Fig. 1a). Individual Pol III subunit occupancies significantly correlate across all comparison groups, as well as with measures of chromatin accessibility, suggesting that ATAC-seq experiments can provide a general prediction of Pol III-transcribed gene activity in the absence of Pol III ChIP experiments (Supplementary Fig. 1b). Overall, the agreement between individual Pol III ChIP experiments suggests that, to a large degree, each subunit accurately reflects the level of Pol III complex gene localization.

The paralogous Pol III subunits POLR3G and POLR3GL are generally expressed at similar levels in human THP-1 monocytes (Supplementary Fig. 1c). Comparison of POLR3G and POLR3GL binding patterns in THP-1 reveals a strong level of overlap in rank normalized signal intensity across genes of all promoter architectures (Supplementary Fig. 1a), consistent with results from previous ChIP-seq experiments in HeLa cells and mouse tissues^50,52^. Individual Pol III complexes have been shown to incorporate either POLR3G or POLR3GL, suggesting overlapping signal densities represent the ability of distinct Pol III complexes to be recruited independent of its subunit composition^52^. However, rank normalized signal intensities do suggest some level of subunit occupancy bias at a subset of genes, most notably where POLR3GL signal is weak, including several tRNA genes, *SNAR-A*, *RPPH1*, and *BCYRN1*, which encodes BC200 RNA (Fig. 1m and Supplementary Fig. 1a). Correlation analysis of each mapped Pol III subunit with either POLR3G or POLR3GL consistently identifies weaker POLR3GL binding at a subset of Pol III-transcribed genes (Supplementary Fig. 1d–i). While POLR3GL ChIP experiments are appropriately enriched for signal at canonical, Pol III-transcribed genes (Supplementary Fig. j), overall weaker signal enrichment intensities in POLR3GL experiments may be due to either biological differences in chromatin-associated levels of POLR3GL in THP-1, or a consequence of weaker antibody affinities for subunit POLR3GL. We, therefore, next sought to integrate Pol III binding profiles with comprehensive maps of Pol III-transcribed small RNA levels and to dissect potential changes in transcription during loss of subunit POLR3G.

### Loss of Pol III complex occupancy at a subrepertoire of genes following differentiation-induced depletion of subunit POLR3G

*POLR3G* gene expression is critical during early development as functional null mutations in *POLR3G* result in embryonic lethality in mice^48,50^. During stem cell differentiation, a gradual loss of *POLR3G* gene expression coincides with an increase in *POLR3GL* mRNA and protein levels, which becomes essential for long-term survival during mouse development^49,50^. Analysis of Pol III subunit gene expression levels across a diverse array of cellular contexts in humans further corroborates the developmental transition in Pol III isoform expression, with high *POLR3G* and low *POLR3GL* gene expression in embryonic stem cells, and conversely low *POLR3G* and high *POLR3GL* gene expression across diverse cell-types and specialized tissues (Supplementary Fig. 2a–c). Like THP-1, both *POLR3G* and *POLR3GL* genes are co-expressed in immortalized cell lines, consistent with previous mapping studies (Supplementary Fig. 2b). These results serve to emphasize that while pluripotent and differentiated cells rely on distinct Pol III complex identities, there are transitional and proliferative contexts in which both subunits POLR3G and POLR3GL are available for Pol III-incorporation and transcription^51,52^.

**Fig. 2:**
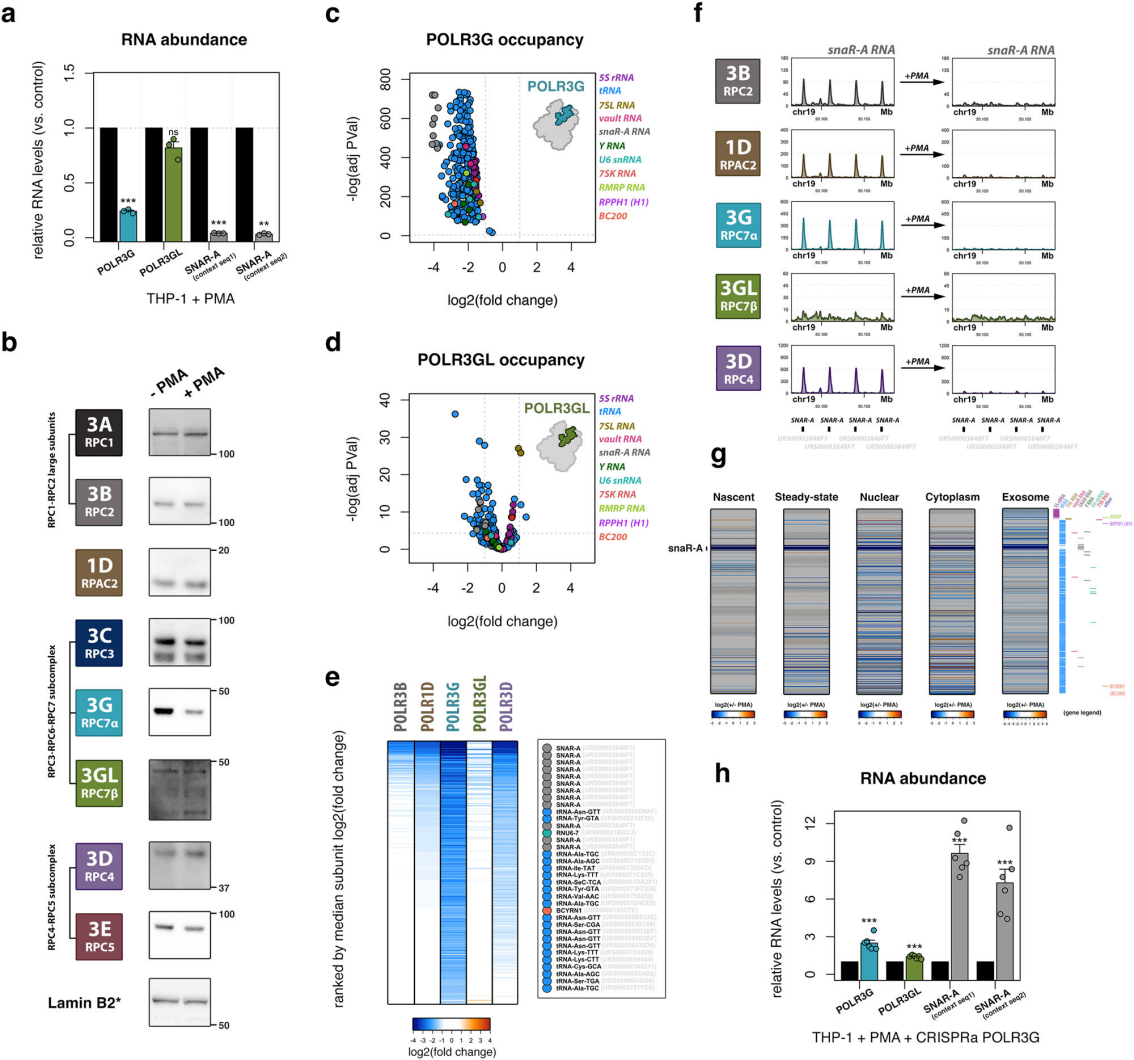
Concomitant loss of POLR3G and Pol III activity modulates cellular and exosomal small RNA repertoires during THP-1 differentiation. **a** RT-qPCR analysis of *POLR3G* and *POLR3GL* mRNA, and snaR-A ncRNA levels following THP-1 differentiation (*n* = 3 biologically independent experiments). *P*-value = 0.00088 (*POLR3G*), 0.099 (*POLR3GL*), 0.00014 (snaR-A seq1), 0.0013 (snaR-A seq2). Significance calculated using Student two-sided *t*-test. **b** Immunoblots for Pol III subunits mapped in THP-1 cells, ±72-h PMA-induced differentiation. *Lamin B2 corresponds to POLR3A and POLR3GL. Observations representative of 2 or more independent experiments. **c**, **d** Volcano plot visualization of dynamic POLR3G (**c**) and POLR3GL (**d**) genomic occupancy ± 72-h PMA-induced differentiation. Gene class indicated by color legend. Significance calculated using edgeR two-sided exactTest function, Benjamini–Hochberg corrected *p*-value. **e** Heatmap visualization of the log2(fold change) of Pol III subunit occupancy for POLR3B, POLR1D, POLR3G, POLR3GL, and POLR3D in THP-1 cells ± 72-h PMA treatment. Heatmap is ordered by median fold change across all subunits. Inset highlights top 35 (10%) of ranked genes. **f** ChIP-seq track visualization for POLR3B, POLR1D, POLR3G, POLR3GL, and POLR3D in THP-1 cells before (left) and after 72-h PMA treatment (right) across a subset of *SNAR-A* genes encoding snaR-A ncRNA. **g** Heatmap visualization of dynamic small RNA profiles in THP-1 monocytes, including nascent, steady-state, nuclear, cytoplasmic, and exosomal small RNA fractions. All heatmaps are ordered by the level of nascent RNA abundance in THP-1 monocytes. Corresponding genes for all heatmaps indicated by color legend on right. **h** RT-qPCR analysis of *POLR3G* and *POLR3GL* mRNA, and snaR-A ncRNA levels following THP-1 differentiation and lentiviral transduction with control or POLR3G-specific CRISPR activation particles (sc-437282 and sc-417072-LAC-2) (*n* = 6 biologically independent experiments). *P*-value = 0.00012 (*POLR3G*), 0.00046 (*POLR3GL*), 5.79e-7 (snaR-A seq1), 4.77e-5 (snaR-A seq2). Significance calculated using Student two-sided *t*-test. Data are presented as mean ± standard error mean (SEM) from the indicated number of independent samples (**a**, **h**). Molecular weights are indicated in kDa. *P ≤ 0.05; **P ≤ 0.01; ***P ≤ 0.001; ns, nonsignificant. Source Data are provided as a Source Data file.

Exposure of THP-1 cells to phorbol 12-myristate 13-acetate (PMA) induces monocyte-to-macrophage differentiation and entry to a quiescent cellular state^57^. The transition of suspension-growing, immature THP-1 monocytes to adherent macrophages lends a simple differentiation system for isolating pure cell populations for genomic experiments^58–60^. Following differentiation, we find that *POLR3G* mRNA levels are significantly downregulated, whereas changes in *POLR3GL* mRNA are comparatively limited (Fig. 2a, Supplementary Fig. 1c). Mirroring changes in gene expression and loss of *POLR3G* mRNA coincides with significant depletion of POLR3G protein levels after 72-h PMA treatment, whereas protein levels for other Pol III subunits remain largely unchanged (Fig. 2b). These data demonstrate a switch in Pol III isoform bias in THP-1 macrophages that is altogether consistent with the dynamic expression patterns observed for POLR3G and POLR3GL in other developmental contexts. ChIP-seq experiments identify robust loss of POLR3G protein occupancy at Pol III-transcribed genes following THP-1 differentiation, confirming that changes in subunit expression bias lead to corresponding changes in Pol III complex identity at the DNA level (Fig. 2c). Whereas POLR3G occupancy is significantly lost across all gene classes, POLR3GL occupancy is marked by significant changes at only a subset of Pol III-transcribed genes, which include examples of both up- and downregulated binding (Fig. 2d). The dynamic gene and protein expression of POLR3G and resulting DNA-binding profiles of POLR3G and POLR3GL show that while proliferative THP-1 monocytes utilize Pol III complexes composed of either subunit, quiescent THP-1 macrophages predominantly rely on Pol III complexes containing POLR3GL.

Analogous genome-wide maps for Pol III subunits POLR3B, POLR1D, and POLR3D uncover loss of ChIP-seq signal across several classes of Pol III-transcribed genes, suggesting Pol III complex occupancy may be disrupted by the loss of POLR3G (Fig. 2e). Ranking genes by the median fold change in subunit occupancy after differentiation identifies a specific subset of Pol III-transcribed genes that exhibit substantial loss in ChIP-seq intensities for POLR3G, POLR3B, POLR1D, and POLR3D (Fig. 2e). Inspection of the top 10% of differentiation-sensitive loci reveals that genes encoding snaR-A, BC200 (*BCYRN1*), and multiple tRNA species are particularly depleted for Pol III localization following PMA treatment. Visual inspection of individual genes marked by diminished Pol III occupancy clearly affirms the loss of subunit localization after 72-h PMA treatment, including at *SNAR-A* genes, tRNA genes, and *BCYRN1* which, again, exhibit comparatively weak POLR3GL ChIP-seq signal prior to treatment with PMA (Fig. 2f and Supplementary Fig. 2d, e).

### Loss of POLR3G restricts transcription and modulates cellular and exosomal small RNA repertoires in THP-1 cells

To characterize the transcriptional and post-transcriptional signatures of THP-1 differentiation, we integrated both nascent and steady-state small RNA profiles in THP-1 monocytes and macrophages. In addition, subcellular fractionation experiments were performed to identify changes in small RNA levels in nuclear, cytoplasmic, and exosomal compartments. THP-1 monocytes feature high levels of nascent 5 S ribosomal RNA, RMRP, 7SL, 7SK, and H1 ncRNA, moderate-to-high levels of nascent vault, snaR-A, U6 spliceosomal, and Y RNA, low abundance of BC200, and a wide range in nascent RNA levels corresponding to Pol III-occupied tRNA genes (Supplementary Fig. 2f). Nuclear and cytoplasmic fractionation experiments appropriately identify nuclear enrichment of RMRP and U6 spliceosomal RNA, and cytoplasmic enrichment of 7SL, snaR-A, and most tRNA species (Supplementary Fig. 2f). Y RNA species dominate THP-1 exosomal small RNA cargo as similarly reported in other contexts (Supplementary Fig. 2f, g)^61,62^. Interestingly, tRNA sequencing reads mapping to tRNA-Lys and tRNA-Gly, which have been recently described to be elevated in plasma exosomes of cancer patients, are also enriched in THP-1 monocyte exosomes (Supplementary Fig. 2f, g)^63^.

Following 72-h PMA treatment, THP-1 macrophages exhibit loss in nascent RNA levels for a specific subset of Pol III-transcribed genes (Fig. 2g). Notably, many Pol III-transcribed genes appear only modestly sensitive or seemingly unaffected in our differentiation system, implying that transitions in Pol III complex identity may not substantially affect transcription at most Pol III-transcribed genes. Nevertheless, changes in nascent RNA are observed for a subrepertoire of the Pol III transcriptome and, in many cases, similarly captured in steady-state and subcellular fractionation small RNA-seq experiments. Dynamic RNA patterns significantly correlate with changes in Pol III occupancy, consistent with the expectation that loss of Pol III recruitment and transcription drive changes in the cellular availability of specific noncoding RNA species after differentiation (Supplementary Fig. 2h). In particular, the most significant changes in nascent and steady-state RNA abundance are attributed to snaR-A ncRNA, consistent with the complete loss of Pol III occupancy identified at the *SNAR-A* gene clusters on chromosome 19 (Fig. 2f). *SNAR-A* downregulation is observed across all fractionation experiments (Fig. 2g), and further confirmed by RT-qPCR after 72-h PMA treatment (Fig. 2a). The temporal dynamics of *SNAR-A* expression closely mirror those of POLR3G protein levels, as POLR3G and snaR-A ncRNA are concomitantly depleted specifically after 72 h (Supplementary Fig. 2i, j). CRISPRa-mediated upregulation and rescue of *POLR3G* gene expression in differentiated THP-1 macrophages results in significant upregulation of snaR-A ncRNA levels, further supporting a putative causal relationship between POLR3G availability and efficient Pol III transcription of *SNAR-A* genes (Fig. 2h, Supplementary Fig. k).

These results altogether demonstrate that monocyte-to-macrophage differentiation is accompanied by a restricted repertoire of Pol III-transcribed genes coinciding with loss of subunit POLR3G. The downregulation of snaR-A ncRNA across all fractionation experiments, including purified exosomes, suggests that dynamic Pol III activity at these genes is broadly influencing both the cellular and extracellular small RNA repertoires in THP-1 cells (Fig. 2g). While fractionation small RNA-seq experiments feature enrichment for snaR-A ncRNA in the cytoplasm, snaR-A is not particularly enriched within the exosomes of THP-1 monocytes compared to total cellular RNA levels (Supplementary Fig. 2f). While this study detects dynamic, exosomal snaR-A ncRNA, whether snaR-A is actively packaged or serves any specific role in circulating extracellular vesicles remains unknown.

### Coordinate loss of POLR3G and a restricted Pol III-transcribed gene repertoire across primary immune cell lineages

To test our model that loss of subunit POLR3G restricts the transcription potential of RNA polymerase III at specific genes, we sought to identify the relationship between Pol III identity and the level of Pol III-transcribed gene activity beyond our THP-1 monocyte-to-macrophage differentiation system. We performed an expansive analysis of Pol III subunit gene expression and Pol III-transcribed gene activity across hematopoietic stem cells and multiple differentiated immune cell lineages, relying on an immense resource of simultaneous gene expression and chromatin accessibility profiling experiments generated within these contexts^64,65^. ATAC-seq, which captures the accessibility of regulatory sequence elements, is also capable of identifying the level of gene accessibility at Pol III-transcribed genes, a unique feature facilitated by the short sequence length of the Pol III transcriptome. Direct comparison of ATAC-seq signal and Pol III subunit occupancy in THP-1 identifies a significant correlation between gene accessibility and Pol III localization, confirming the utility of ATAC-seq as a general indicator of Pol III occupancy absent direct ChIP-seq experiments against Pol III subunits (Supplementary Fig. 1b). Thus, gene accessibility analysis offers an advantageous large-scale approach for directly querying dynamic gene signatures associated with Pol III transcription, while also cutting through the complex mixture of nascent, intermediate, and processed mature small RNA species captured in steady-state RNA experiments.

**Fig. 3:**
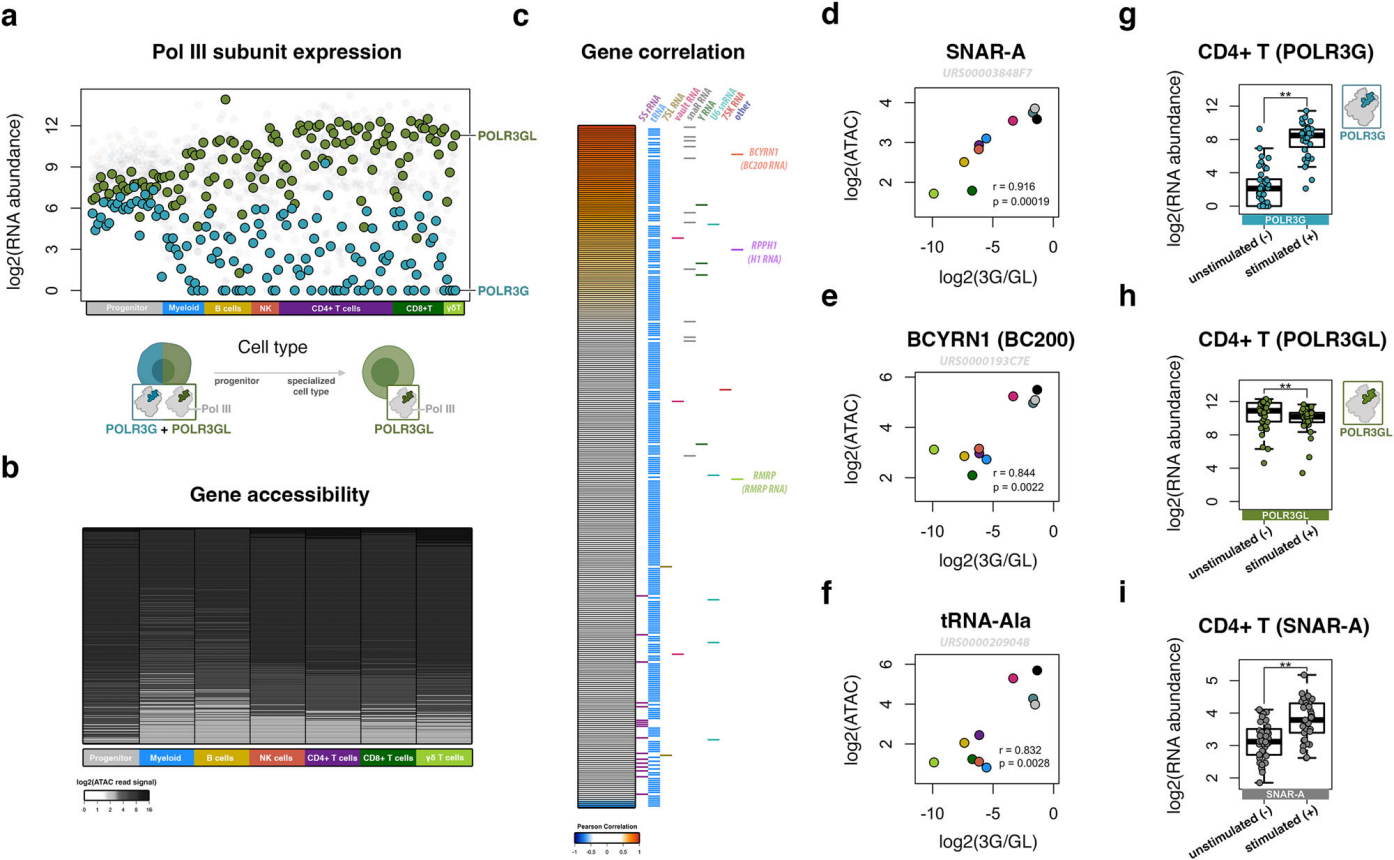
Primary immune cell differentiation is marked by loss of *POLR3G* gene expression and restricted chromatin features at a subset of genes, including *SNAR-A*. **a** Pol III subunit gene expression profiles across hematopoietic progenitor cells and distinct differentiation immune cell lineages, including myeloid, B cell, natural killer (NK), CD4 + T, CD8 + T, and γδ T cells, with emphasis on *POLR3G* and *POLR3GL*. **b** Pol III-transcribed gene accessibility (ATAC-seq) across hematopoietic progenitor cells and distinct differentiation immune cell lineages corresponding to Fig. 3a. Individual rows (genes) are ranked by median accessibility score across cell types. Loss of gene accessibility is signified by weakened ATAC-seq signal across a subset of genes in differentiated immune cell populations. **c** Heatmap visualization of correlations between gene accessibility and gene expression ratio of *POLR3G:POLR3GL*. Correlation represents integration of average context-specific expression level (Fig. 3a) and average context-specific gene accessibility (Fig. 3b). Corresponding genes indicated by color legend on right. **d**–**f** Individual correlation profiles of *SNAR-A* (**d**), *BCYRN1* (**e**), and tRNA-Ala gene (URS0000209048) chromatin accessibility with *POLR3G:POLR3GL* gene expression ratios in primary immune cells depicted in heatmap (Fig. 3c). Pearson’s correlation, corresponding *p*-values computed as two-sided test. **g** *POLR3G* gene expression profile in primary immune CD4 + T cells before and after co-stimulation with anti-CD3/CD28 Dynabeads^65^. From left to right, *N* = 37,40 independent experiments. Statistical analysis with a two-sided Wilcoxon rank-sum test. *P* = 2.3e-11. **h** *POLR3GL* gene expression profile in primary immune CD4 + T cells before and after stimulation. From left to right, *N* = 37, 40 independent experiments. Statistical analysis with a two-sided Wilcoxon rank-sum test. *P* = 0.0047. **i** *SNAR-A* gene accessibility profile in primary immune CD4 + T cells before and after stimulation. From left to right, *N* = 40,37 independent experiments. Statistical analysis with a two-sided Wilcoxon rank-sum test. *P* = 2.56e-5. Box plot center lines correspond to median, lower and upper hinges first and third quartile. Whiskers present minimum and maximum values not exceeding 1.5*IQR beyond first and third quartile. *P ≤ 0.05; **P≤ 0.01; ***P ≤ 0.001; ns, nonsignificant. Source Data are provided as a Source Data file.

Analysis of Pol III subunit gene expression across a multitude of primary human immune cell types again captures the differentiation-associated loss of *POLR3G* gene expression and upregulation of *POLR3GL* mRNA (Fig. 3a, Supplementary Fig. 3a, b). Whereas hematopoietic stem cells and progenitor cells express both *POLR3G* and *POLR3GL*, differentiated immune effector cells, including subsets of B cells, natural killer (NK) cells, CD4 + T cells, CD8 + T cells, and γδ T cells typically express high levels of *POLR3GL* and limited-to-absent levels of *POLR3G* mRNA. Analogous gene expression profiling in immune cells isolated from patients with acute myeloid leukemia (AML) identifies co-expression of both *POLR3G* and *POLR3GL* mRNA in pre-leukemic hematopoietic stem cells (pHSCs), leukemia stem cells (LSC), and leukemic blast cells (Blast) (Supplementary Fig. 3a). The THP-1 cell line, which expresses both *POLR3G* and *POLR3GL*, was derived from an acute monocytic leukemia patient and likely represents an immature precursor monocyte; thus the expression profile appears to appropriately mirror that of leukemic blast and immune progenitor cells^64^. The co-expression of *POLR3G* and *POLR3GL* in primary hematopoietic progenitor cells also serves to confirm the relevancy of contexts in which POLR3G and POLR3GL are simultaneously available for incorporation into the Pol III complex.

Given the significant loss of *POLR3G* gene expression within differentiated immune cell lineages, we next queried the level of gene accessibility across the Pol III transcriptome in all tested immune cell contexts. In comparison with the ATAC-seq profile in immune progenitor cells, all differentiated immune cell lineages exhibit a loss of gene accessibility at a specific subset of Pol III-transcribed genes (Fig. 3b). This feature is largely absent from the chromatin profile in pHSC, LSC, and Blast cells, where *POLR3G* gene expression is only modestly decreased in leukemic blast cells (Supplementary Fig. 3c). Overall, the diminishing accessibility of specific Pol III-transcribed genes coinciding with loss of *POLR3G* gene expression is broadly consistent with our model derived from experiments in THP-1, in which depletion of *POLR3G* in turn diminishes Pol III activity at specific genes. We, therefore, next asked whether POLR3G-sensitive genes in THP-1 cells are also broadly downregulated in primary immune cell populations.

### A subrepertoire of Pol III-transcribed genes, including *SNAR-A*, correlate with *POLR3G* gene expression bias across immune cell lineages

The measurement of both Pol III subunit gene expression and downstream gene accessibility across a multitude of immune cell types allows for direct comparison of predicted gene activity with *POLR3G* gene expression. We specifically considered the ratio of *POLR3G*:*POLR3GL* gene expression, which accounts for the relationship between these two subunits and potential biases that arise due to higher levels of a specific subunit (Supplementary Fig. 3d). Strong correlations are observed between the chromatin signatures at a subset of Pol III-transcribed genes and the level of *POLR3G* gene expression bias in both individual primary immune samples and aggregate cell type analyses (Fig. 3c–f and Supplementary Fig. 3e–i). Visualization of the correlation scores between gene accessibility and *POLR3G:POLR3GL* gene expression ratios in aggregate cell populations again identifies a subrepertoire of Pol III-transcribed genes that appear to be modulated by POLR3G subunit availability (Fig. 3c). Consistent with observations during THP-1 differentiation, *SNAR-A*, *BCYRN1*, and a subset of tRNA genes are among the cohort of genes with correlative chromatin features in primary immune cell populations (Fig. 3c). Direct visualization of individual genes highlights this relationship: gene accessibility is low in differentiated immune cell lineages and high in progenitor and leukemia-related contexts with high *POLR3G* gene expression (Fig. 3d–f and Supplementary Fig. 3g–i). Notably, the chromatin signatures at most Pol III-transcribed genes do not correlate with the dynamic identity shift from POLR3G to POLR3GL across cell populations (Fig. 3c). Visualization of *RMRP*, for example, highlights an example of Pol III transcription that, like most Pol III-transcribed genes, appears entirely unperturbed by the transition in Pol III complex identity favoring subunit POLR3GL (Supplementary Fig. 3j).

Stimulation of primary immune B and T cells, which induces cell proliferation, results in widespread changes in gene expression and chromatin accessibility^65^. *POLR3G* gene expression is upregulated in B and T cells following robust stimulation, whereas *POLR3GL* is comparatively stable (Fig. 3g, h and Supplementary Fig. 3k–n). Consistent with POLR3G rescue experiments in THP-1 macrophages, we find that *SNAR-A* gene accessibility mirrors the level of *POLR3G* upregulation before and after stimulation: significant increases in *SNAR-A* chromatin features are identified in CD4+ and CD8 + T cells, where upregulation of *POLR3G* is most robust (Fig. 3i and Supplementary Fig 3o). *SNAR-A* gene accessibility also increases in stimulated B cells, although these changes do not reach statistical significance (Supplementary Fig. 3p). Overall, these results show that upregulation of POLR3G re-establishes activity at *SNAR-A* genes, providing further evidence that *SNAR-A* genes represent a specialized subrepertoire of Pol III-transcribed genes enhanced by POLR3G and its corresponding Pol III complex identity.

### Cancer-associated POLR3G upregulation correlates with dynamic chromatin features and poor survival outcomes

**Fig. 4:**
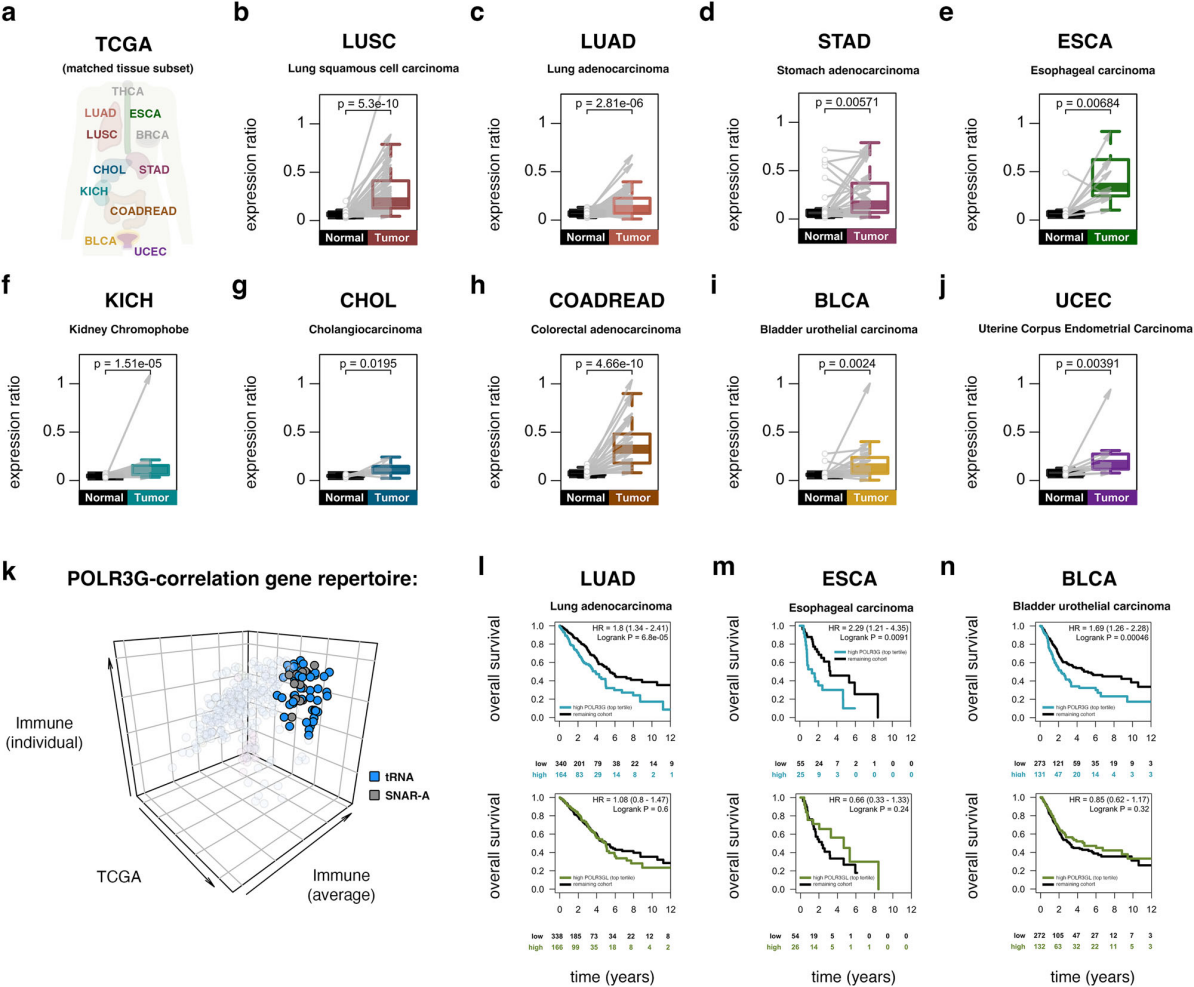
Re-establishment of POLR3G and downstream chromatin signatures in human primary solid tumors and poor survival outcomes in patients with POLR3G overexpression. **a** Diagram of TCGA patient-matched normal and primary solid tumor cancer types profiled for *POLR3G* and *POLR3GL* gene expression levels in this study. **b**–**j** Gene expression ratios of *POLR3G/POLR3GL* in lung squamous cell carcinoma (**b**, *n* = 51), lung adenocarcinoma (**c**, *n* = 58), stomach adenocarcinoma (**d**, *n* = 32), esophageal carcinoma (**e**, *n* = 11), kidney chromophobe (**f**, *n* = 25), cholangiocarcinoma (**g**, *n* = 9), colorectal adenocarcinoma (**h**, *n* = 32), bladder urothelial carcinoma (**i**, *n* = 19), and uterine corpus endometrial carcinoma (**j**, *n* = 10). Gray arrows represent individual patient-matched normal and primary solid tumors. Corresponding *p*-values determined using paired Wilcoxon signed-rank two-sided test. Box plot center lines correspond to median, lower and upper hinges first and third quartile. Whiskers present minimum and maximum values not exceeding 1.5*IQR beyond first and third quartile. **k** 3D scatterplot visualization of the subrepertoire of Pol III-transcribed genes with correlative *POLR3G* and predicted gene activity in The Cancer Genome Atlas (TCGA) and primary immune profiling experiments. **l**–**n** Kaplan–Meier analysis of overall survival of TCGA donors stratified by high *POLR3G* gene expression (top tertile; top panels) or high *POLR3GL* gene expression (top tertile; bottom panels) in lung adenocarcinoma (**l**), esophageal carcinoma (**m**), and bladder urothelial carcinoma (**n**). Corresponding *p*-values determined using log-rank two-sided test; HR = hazard ratio risk of dying. Source Data are provided as a Source Data file.

The re-establishment of POLR3G and *SNAR-A* chromatin features in stimulated B and T cell populations, which exit quiescence and re-enter a proliferative cellular state^65–67^, mirrors the context-specific expression of *POLR3G* in proliferative stem cells and upregulation in immortalized cell lines. *POLR3G* gene expression is also upregulated in specific cancer contexts, such as transitional cell carcinoma^68^. Building on the putative POLR3G-sensitive repertoire in THP-1 and primary immune cells, we sought to profile Pol III identity and downstream activity in cancer contexts. The Cancer Genome Atlas (TCGA), a database of clinical and integrated molecular signatures collected from primary human cancer tissues, provides an invaluable resource for exploring both dysregulated gene activity and chromatin features in a multitude of cancer contexts^69,70^. Analysis of *POLR3G* and *POLR3GL* expression levels in these data uncover significant increases in *POLR3G* gene expression bias in several primary solid tumors when compared to patient-matched normal tissues, including lung squamous cell carcinoma, lung adenocarcinoma, stomach adenocarcinoma, esophageal carcinoma, kidney chromophobe, cholangiocarcinoma, colorectal adenocarcinoma, bladder urothelial carcinoma, and uterine corpus endometrial carcinoma (Fig. 4a–j). Notably, patient-matched breast, thyroid, and prostate primary cancer tissues are characterized by less, or insignificantly higher *POLR3G* bias, suggesting *POLR3G* dysregulation may be less common in these and potentially other contexts (Supplementary Fig. 4a–c).

Integrated analysis of *POLR3G* and *POLR3GL* gene expression with predictive chromatin features in individual tumor samples once again identifies positive correlations between *SNAR-A* and *POLR3G* expression, further extending this relationship beyond THP-1 and primary immune cells into cancer contexts (Fig. 4k, Supplementary Fig. 4d, e). Inspection of Pol III-transcribed genes with positive correlation scores in both cancer and primary immune differentiation contexts identifies *SNAR-A* as well as a subset of tRNA genes (Fig. 4k, Supplementary Fig. 4f). Overall, this subrepertoire of Pol III-transcribed genes, identified only through correlative chromatin features in immune cells and primary solid tumors, is more significantly downregulated during THP-1 differentiation with respect to Pol III occupancy, nascent RNA, and total, nuclear, and cytoplasmic steady-state RNA levels compared to noncorrelative genes (Fig. 4g–k). In the case of Pol III occupancy, nascent, and nuclear small RNA profiles, which more accurately reflect dynamic transcription patterns, this significance is independent of *SNAR-A* representation, further suggesting that the expression of specific tRNA genes is likely enhanced by subunit POLR3G (Supplementary Fig. 4l–p).

The biological significance of *POLR3G* upregulation and downstream activities is made evident by Kaplan–Meier analyses, which identify adverse outcomes for patients with tumors expressing elevated levels of *POLR3G* mRNA in a myriad of cancer contexts. For example, high *POLR3G* gene expression identifies a subgroup of patients with unfavorable survival outcomes in lung adenocarcinoma, esophageal carcinoma, bladder urothelial carcinoma, kidney renal clear cell carcinoma, uterine corpus endometrial carcinoma, and liver hepatocellular carcinoma (Fig. 4l–n, Supplementary Fig 4q–s; low patient count (n = 80) in esophageal carcinoma cohort). This relationship is not observed in contexts where *POLR3G* upregulation is less common (breast invasive carcinoma and thyroid carcinoma, Supplementary Fig. 4t, u), together suggesting Pol III transcription dysregulation may be a pronounced feature in a multitude but subset of cancer contexts. In all contexts, high *POLR3GL* gene expression is not an indicator of adverse outcomes. These results serve to emphasize the overall significance of POLR3G and Pol III identity as a potential disease factor and may indicate that contexts featuring POLR3G-enhanced transcription are at risk of aggressive growth, metastasis, and unfavorable outcomes.

### Subunit-specific disruption of POLR3G induces rapid loss of snaR-A ncRNA and specific tRNA species

The Pol III-specific inhibitor, ML-60218, was discovered as an analog of small molecule inhibitors of Pol III transcription in *S. cerevisiae* with high potency in human cells^71^. Recent structural modeling suggests the target site of ML-60218 may reside within the trigger loop helix at the active center of RNA polymerase III, a site proximal to the bridge helix of POLR3A (RPC1) and conserved regions of an autoinhibitory motif in the C-terminal tail of POLR3G^72^. ML-60218 exposure causes POLR3G depletion and a switch from POLR3G enrichment towards POLR3GL enrichment in coimmunoprecipitation experiments with other Pol III subunits^73^. Though the mechanism of POLR3G-sensitivity to ML-60218 requires further investigation, the overlapping effects reported for *POLR3G* knockdown and ML-60218 exposure help to explain why ML-60218 fails to effectively inhibit Pol III transcription in specific contexts, such as those recently reported during preadipocyte-to-adipocyte differentiation^30^. Instead, ML-60218 is likely an effective strategy for inhibiting Pol III transcription in early developmental windows and other contexts with high POLR3G abundance.

**Fig. 5:**
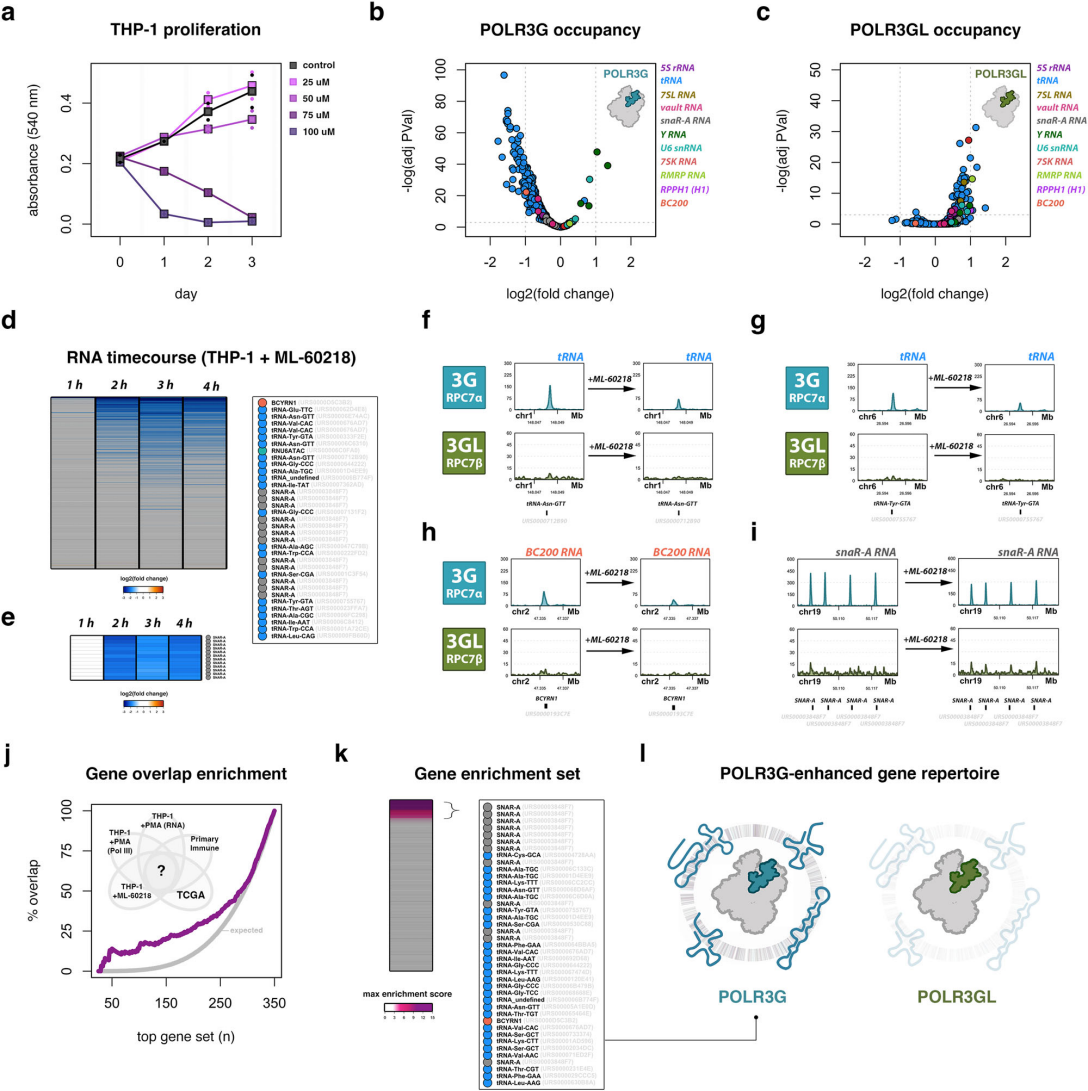
Subunit-specific drug inhibition of POLR3G induces rapid loss of POLR3G occupancy and snaR-A ncRNA abundance. **a** THP-1 growth curves were generated using MTT cell proliferation assays in cells exposed to 0 (control), 25, 50, 75, and 100 uM ML-60218. **b**, **c** Volcano plot visualization of dynamic POLR3G (**b**) and POLR3GL (**c**) genomic occupancy in THP-1 cells ± 4 h exposure to Pol III inhibitor ML-60218 (25 uM). Gene class indicated by color legend. Significance calculated using edgeR two-sided exactTest function, Benjamini–Hochberg corrected *p*-value. **d** Heatmap of log2(fold change) in small RNA abundance for Pol III-transcribed genes in THP-1 cells at 0, 1, 2, 3, and 4 h post-exposure to Pol III inhibitor ML-60218 (25 uM). Heatmap is ordered by the average gene log2(fold change) across the timecourse experiment. Inset highlights the top 35 (10%) dynamic genes. **e** Heatmap subset from timecourse experiment (panel d) for *SNAR-A* genes. **f**–**i** ChIP-seq track visualization for POLR3G and POLR3GL in THP-1 cells before (left) and after 4h ML-60218 exposure (right) at example tRNA genes *tRNA-Asn-GTT* (URS0000712B90) (**f**) and *tRNA-Tyr-GTA* (URS0000755767) (**g**), *BCYRN1* (**h**), and *SNAR-A* genes (**i**). **j** Moving Venn-diagram overlap analysis of ML-60218 gene sensitivity 4 h post-exposure with diverse experimental results, including dynamic Pol III occupancy (THP-1 +PMA; Fig. 2), dynamic RNA abundance (THP-1 +PMA; Fig. 2), *POLR3G* and gene accessibility correlation in primary immune cell differentiation (aggregate cell type analysis; Fig. 3), and *POLR3G* and gene accessibility correlation in primary solid tumors (TCGA; Fig. 4). **k** Heatmap visualization of maximum enrichment scores, related to Fig. 5j, for individual Pol III-transcribed genes. Inset highlights the subrepertoire of Pol III-transcribed genes that are most likely to be enhanced by subunit POLR3G (maximum enrichment score > = 3). **l** Model illustration that, compared to POLR3GL, subunit POLR3G enhances the expression of genes encoding specific small noncoding RNA species, including snaR-A, specific tRNAs, and likely BC200 (*BCYRN1* gene). Source Data are provided as a Source Data file.

We explored the effects of ML-60218 on THP-1 cells as an additional, complementary approach to further dissect the contribution of POLR3G to Pol III transcription potential in our system. THP-1 cells exhibit a narrow range of ML-60218 sensitivity, with limited levels of growth disruption observed at 25–50 uM, and loss of cell viability at 75–100 uM (Fig. 5a). Exposure of THP-1 to 25 uM ML-60218 results in rapid loss of POLR3G localization at most Pol III-transcribed genes within 4 h (Fig. 5b). POLR3GL protein occupancy is comparatively stable in the presence of ML-60218, with some level of upregulation (Fig. 5c), confirming subunit-specific or strongly biased disruption of subunit POLR3G following ML-60218 exposure. These results are consistent with previous characterizations of ML-60218 disruption, and highlight the utility of ML-60218 as a potent tool for disrupting POLR3G activity.

Analogous profiling of small RNA abundance in THP-1 monocytes before and after 4-h ML-60218 exposure identifies significant loss in RNA levels for a specific subset of Pol III-transcribed genes, including *SNAR-A* (Supplementary Fig. 5a). Temporal profiling of small ncRNA at 1, 2, 3, and 4 h post ML-60218 exposure identifies substantial changes within 2 h of drug exposure, demonstrating a rapid effect of POLR3G disruption on the RNA abundance of a subrepertoire of Pol III-transcribed genes (Fig. 5d, e). Inspection of POLR3G and POLR3GL subunit occupancies at ML-60218 sensitive Pol III-transcribed genes once again illustrates the loss of POLR3G localization at *SNAR-A* and other genes with low levels of POLR3GL, with seemingly insufficient increases in POLR3GL localization to account for POLR3G disruption (Fig. 5f–i).

The subrepertoire of Pol III-transcribed genes sensitive to ML-60218 exposure strongly overlap genes downregulated by POLR3G depletion during THP-1 differentiation (Supplementary Fig. 5b, c). Genes disrupted after 4 h ML-60218 treatment are more significantly downregulated with respect to Pol III occupancy, nascent, steady-state, nuclear, and cytoplasmic RNA levels during THP-1 differentiation, in all cases independent of *SNAR-A* gene representation (Supplementary Fig. 5d–m). Integration of ML-60218 gene sensitivities in THP-1 with the dynamic Pol III and ncRNA signatures observed during THP-1 differentiation, and the correlative POLR3G-chromatin features identified in primary immune and primary solid tumors, uncovers strong gene overlap enrichment across these experiments (Fig. 5j). The spectrum of individual gene representation and overlap enrichment scores derived from this analysis serves to clarify the POLR3G-enhanced Pol III transcriptome, which includes snaR-A and BC200 ncRNA and several tRNA species (Fig. 5k, l).

Included in the list of POLR3G-enhanced genes is *BCYRN1*, which is also among the top dynamic small RNA gene signatures identified following ML-60218-induced POLR3G disruption (Fig. 5d). However, changes in BC200 ncRNA levels do not reach statistical significance, likely due to the limited expression and read count enrichment for BC200 in steady-state small RNA profiling experiments (Supplementary Fig. 5a). Nevertheless, loss of POLR3G occupancy at *BCYRN1* is significant, and visualization of ChIP-seq signal tracks illustrates the dynamic POLR3G localization as similarly observed during THP-1 differentiation (Fig. 5b, h). Although *BCYRN1* was not identified as a significant correlative feature with *POLR3G:POLR3GL* gene expression ratios in cancer, it was among the strongest correlative features in primary immune cells, and otherwise ranks among the top cohort with respect to concordant patterns in multiple experiments (Fig. 5k). These results suggest that, like *SNAR-A*, expression of *BCYRN1* may also be sensitive to POLR3G availability, but that additional factors may be required to increase the expression and/or post-transcriptional stability of BC200 RNA in specific contexts.

### Cancer-associated Pol III identity and downstream transcription potential is driven by MYC

**Fig. 6:**
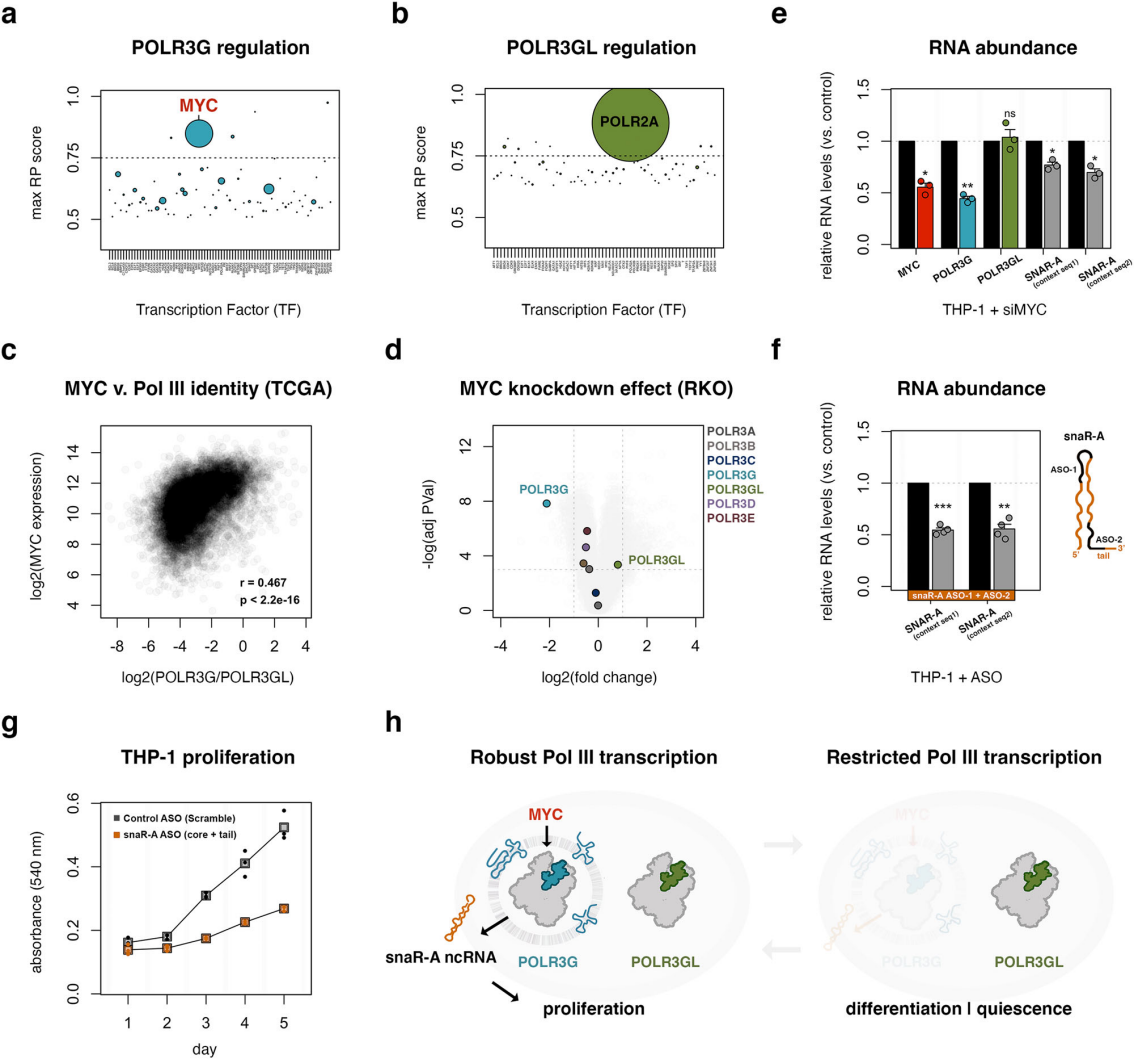
MYC promotes *POL*R3G gene expression, shaping Pol III identity and downstream transcription activities associated with cell proliferation. **a**, **b** Maximum cistromeDB regulatory potential (RP) scores for transcription factors likely to regulate *POLR3G* (**a**) and *POLR3GL* (**b**) gene expression. Point size corresponds to number of experiments with top RP score. **c** Correlation analysis of *MYC* expression and *POLR3G/POLR3GL* gene expression ratios in TCGA samples. Spearman’s rank correlation, corresponding *p*-value computed as two-sided test. *P* < 2.2e-16. **d** Volcano plot visualization of mRNA changes following robust knockdown of *MYC* in RKO cells (Topham et al., 2015)^82^, highlighting *POLR3G*, *POLR3GL*, and Pol III subunits mapped in THP-1 cells. **e** RT-qPCR analysis of *MYC*, *POLR3G*, and *POLR3GL* mRNA, and snaR-A ncRNA levels following *MYC* KD (*n* = 3 biologically independent experiments). *P*-value = 0.014 (*MYC*), 0.0037 (*POLR3G*), 0.70 (*POLR3GL*), 0.017 (snaR-A seq1), 0.018 (snaR-A seq2). Significance calculated using Student two-sided *t*-test. **f** RT-qPCR analysis snaR-A ncRNA levels in THP-1 cells transfected with ASOs targeting conserved sequences within the core (ASO-1) and tail (ASO-2) regions of snaR-A, 48 h post transfection (*n* = 4 biologically independent experiments). *P*-value = 0.00051 (snaR-A seq1), 0.0053 (snaR-A seq2). Significance calculated using Student two-sided *t*-test. Data are presented as mean ± standard error mean (SEM) from the indicated number of independent samples (**e**, **f**). *P ≤ 0.05; **P ≤ 0.01; ***P ≤ 0.001; ns, nonsignificant. **g** THP-1 growth curves were generated using MTT cell proliferation assays in THP-1 cells following transfection with control (scramble) or ASO-1 and ASO-2 targeting snaR-A ncRNA. **h** Model: MYC-driven expression of *POLR3G* shapes Pol III identity, the availability of subunit POLR3G enhances Pol III activity and transcription potential, downstream expression of snaR-A ncRNA promotes cell viability and proliferation. Differentiation and quiescence are characterized by concomitant loss of MYC, POLR3G, and snaR-A; Pol III transcription potential is thereby restricted in contexts limited to subunit POLR3GL. Source Data are provided as a Source Data file.

Previous studies have identified *POLR3G*-specific promoter localization of transcription factor MYC, suggesting MYC regulates the expression *POLR3G*, but not *POLR3GL*^52^. In further support of this finding, cistromeDB analysis of regulatory potential scores against an assemblage of transcription factor ChIP-seq experiments assigns a strong prediction score for MYC-specific regulation of the *POLR3G* promoter region (Fig. 6a)^74–76^. MYC is not among the predicted regulatory factors at the *POLR3GL* promoter, which is instead enriched for POLR2A binding, a generic signature of Pol II transcription activity and potential indicator of promoter-proximal pausing regulation of the *POLR3GL* gene (Fig. 6b). MYC is a well-established driver of transcriptional amplification; MYC overexpression results in enhanced promoter occupancy and increased transcriptional output of existing gene expression programs^77^. MYC is also a central regulator of metabolic reprogramming and proliferation of stimulated B and T cell populations^78–80^, and a well-established oncogene driving a multitude of cancers^81^, suggesting MYC might underlie the dynamic Pol III identity signatures observed within these contexts.

Comparison of MYC and Pol III identity biases in TCGA data identifies a significant relationship between *MYC* gene expression and *POLR3G/POLR3GL* gene expression ratios across tumor and non-tumor samples, supporting a model in which Pol III identity is modulated by MYC-driven transcription amplification in cancer contexts (Fig. 6c). Direct functional evidence for this relationship comes from genome-wide measures of RNA levels following robust knockdown of *MYC* in proliferating RKO cells, a poorly differentiated colon carcinoma cell line, a context with direct relevance to Pol III identity dysregulation within colon and rectal adenocarcinomas^82^. *POLR3G* gene expression levels, which are otherwise elevated in RKO cells, are significantly diminished in response to *MYC* RNAi, but not in response to non-targeting siRNA or similar knockdown experiments of unrelated factors *KCNK1* and *SNTA1* (Fig. 6d, Supplementary Fig. 6a). *POLR3GL* mRNA, in contrast, increases moderately following *MYC* knockdown, overall suggesting a significant shift in Pol III identity dependent on MYC transcription factor activity (Supplementary Fig. 6b). Knockdown of MYC in this system has a comparatively modest effect on the mRNA abundance of other Pol III subunits, signifying a special role for MYC in modulating *POLR3G* gene expression and downstream Pol III activity (Fig. 6d).

We find that MYC also acts as a regulatory driver of *POLR3G* gene expression in THP-1 cells, with connections to downstream transcription potential. First, *MYC* mRNA levels significantly decrease in THP-1 macrophages following 72-h PMA treatment, suggesting the observed shift in Pol III identity may be driven by MYC availability and regulation of *POLR3G* gene expression (Supplementary Fig. 6c). As reported in other contexts, direct knockdown of *MYC* in THP-1 monocytes results in significant depletion of *POLR3G* mRNA (Fig. 6e, Supplementary Fig. 6d). This effect is specific for *POLR3G*, with no observable change in *POLR3GL* mRNA levels in our system. In contrast, snaR-A RNA levels also decrease following *MYC* knockdown in THP-1 cells, coinciding with loss of *MYC* and *POLR3G* mRNA levels (Fig. 6e). These results confirm subunit-specific gene regulation of *POLR3G*, and establish a link between MYC, POLR3G, and the POLR3G-enhanced repertoire of Pol III-transcribed genes in THP-1 cells.

The connections between *MYC*, *POLR3G*, and cell proliferation and cancer raise important questions about the significance of POLR3G-enhanced transcription, generally, and the functional activities of snaR-A ncRNA, specifically. We tested the ability of antisense oligonucleotides (ASOs), directed at conserved sequences within the core and tail regions of snaR-A ncRNA, to disrupt snaR-A levels and examined the downstream consequences of snaR-A disruption on cell proliferation in THP-1 monocytes. snaR-A ncRNA levels are measurably diminished by both ASO designs, though most significantly when targeting both the core and tail regions of snaR-A (Fig. 6f). THP-1 cell growth is indeed sensitive to snaR-A-targeting ASOs compared to scramble control ASOs (Fig. 6g), supporting a role for snaR-A in cell proliferation and consistent with previously reported effects of snaR-A depletion on cell proliferation in breast cancer cells^27,83^. The disruption of THP-1 growth indicates that modulation of POLR3G availability and downstream transcription potential has important, functional consequences, and suggests snaR-A ncRNA may play an underappreciated role in promoting the viability and proliferation of immortalized THP-1 cells and other cancer contexts.

## Discussion

Using a combination of in vitro differentiation and subunit disruption experiments, as well as large-scale analysis of simultaneous gene expression and chromatin signatures, our results strongly suggests that POLR3G and, consequently, Pol III identity itself, modulate transcription potential with significant implications for development and disease. We find that *SNAR-A* gene activity consistently tracks with *POLR3G* gene expression levels in primary immune cell populations and cancer tissues and represents the dominant dynamic feature of both THP-1 differentiation and POLR3G disruption experiments. While this study is not the first to simultaneously map POLR3G and POLR3GL protein occupancy and transcription, it does not appear that the *SNAR-A* gene has been previously considered in other Pol III occupancy studies. Further, the *SNAR-A* gene is evolutionarily restricted to Hominidae^21^, and would therefore not be identified in studies of non-Hominidae vertebrate species. The concomitant upregulation of *POLR3G* mRNA and rescue of snaR-A ncRNA levels in differentiated THP-1 macrophages, and analogous re-establishment of *POLR3G* gene expression and active *SNAR-A* gene accessibility features in stimulated primary immune cells provides compelling evidence that POLR3G may play a unique role in establishing competent and perhaps efficient transcription at these loci.

The discovery of POLR3G-driven expression of *SNAR-A* genes and the expanded Pol III gene repertoire in proliferating cells demands a better understanding of the mechanism underlying POLR3G-enhanced transcription. The enrichment of POLR3G signal compared to POLR3GL at *SNAR-A* and other genes suggests that Pol III complexes incorporating subunit POLR3G may be recruited and/or establish competent transcription at these loci with greater efficiency compared to Pol III complexes with POLR3GL. Perhaps noteworthy is the modest increase in POLR3GL signals at *SNAR-A* genes observed immediately following rapid ML-60218 disruption of POLR3G (Fig. 5i), suggesting POLR3GL-incorporated Pol III complexes are not incapable of recruitment at these loci. However, the significant loss of snaR-A ncRNA following POLR3G disruption again suggests that POLR3GL is likely ineffective and/or inefficient at driving transcription at these and other genes. In such a case, POLR3G would likely outcompete POLR3GL occupancy and activity, explaining the enrichment of POLR3G occupancy in THP-1 monocytes and increase in POLR3GL signal at these genes only following POLR3G disruption.

The comparatively weaker POLR3GL signal at Pol III-transcribed genes in THP-1 cells raises additional questions about competitive incorporation, recruitment, and transcription in contexts featuring both POLR3G and POLR3GL and potential noncanonical activities in circumstances with high POLR3G and/or POLR3GL stoichiometries. For example, the complete loss of Pol III complex localization and lack of POLR3GL occupancy at *SNAR-A* genes following THP-1 differentiation leads us to an additional speculative model: POLR3G may localize to the *SNAR-A* gene cluster independent of the full Pol III machinery, potentially via RPC3-RPC6-RPC7 subcomplexes through interactions with TFIIIB. Recruitment might conceivably establish an accessible chromatin landscape that facilitates subsequent recruitment of Pol III for transcription. Potential supporting evidence for this model includes the comparatively moderate disruption of POLR3G occupancy at *SNAR-A* genes after 4 h ML-60218 exposure (Fig. 5b, i), despite strong disruption of snaR-A ncRNA levels at the same time point (Fig. 5d, e). One interpretation of this result is that ML-60218 disrupts Pol III:POLR3G incorporation but does not disrupt POLR3G-independent gene localization. A second interpretation is that, following ML-60218 disruption, POLR3GL more effectively outcompetes POLR3G at genes in which efficient transcription can be established. Other potential mechanisms include the possibility, based on recent structural data, that POLR3G rescues the activity of specific genes from MAF1-mediated repression, implying unique biases or efficiencies of MAF1 repression directed at specific genes.

The contrasting clinical significance of *POLR3G* and *POLR3GL* gene upregulation in cancer contexts, wherein high *POLR3G* gene expression is associated with poor survival outcomes and high *POLR3GL* is not, strongly hints at important functional differences between POLR3G and POLR3GL and exposes POLR3G-driven transcription as a potential disease factor. Our study identifies *SNAR-A* expression as an important element in this dichotomy, and we speculate that other differences may also be identified. While studies of snaR-A ncRNA are currently limited, *SNAR-A* has been shown to be upregulated in hepatocellular and ovarian carcinoma and HER2-positive breast cancer cells and, like POLR3G, immortalized cell lines^25–27^. A snaR-A-derived noncanonical miRNA, which targets NME-1, was recently identified and shown to promote cancer cell migration, suggesting a potential link between *SNAR-A* expression, cancer proliferation, and metastasis^84^. However, full-length snaR-A ncRNA is required for the observed growth effects, as snaR-A-miRNA alone does not promote cell proliferation. Instead, full-length snaR-A most likely modulates the activity of ILF3, the RNA-binding protein to which it is known to interact^22,85^. ILF3 also promotes cell proliferation and, like POLR3G, is an unfavorable prognostic indicator across multiple cancers^86^. Future experiments dissecting the function of snaR-A ncRNA and its potential modulation of ILF3 activity will help to further understand the consequence of the POLR3G-enhanced transcription program.

Finally, our study confirms subunit-specific regulation of *POLR3G* gene expression by MYC, connecting top-level regulation of Pol III identity to downstream transcription potential in the form of snaR-A ncRNA with implications for cell proliferation (Fig. 6h). BC200 ncRNA, which also presents evidence of gene-level correlation with POLR3G availability, has been similarly reported to be enhanced by MYC and essential for viability and proliferation in cancer contexts^23,24,87–90^. In addition to snaR-A and BC200, we find that specific tRNA genes are sensitive to POLR3G disruption, including tRNA-Ala genes recently shown to be sensitive to MYC depletion in human HEK293 cells^91^. In light of the POLR3G-enhanced transcriptome reported here, previous MYC-enhanced or MYC-sensitive Pol III activities reported likely included modulation of Pol III identity and downstream transcription potential. Like POLR3G, high levels of MYC expression are associated with poor survival outcomes in specific and overlapping cancer contexts (Supplementary Fig. 6f–l), evidence of concurrent MYC and Pol III dysregulation. While the analogous regulatory features underlying POLR3GL expression remain to be identified, the concurrent loss of MYC and POLR3G appears to be a sufficient mechanism for restricting transcription potential in differentiated and quiescent cellular contexts. We speculate that POLR3GL itself may play an equally important role in placing functional limits on Pol III transcription, the mechanisms and significance of which remain exciting areas for future research.

## Methods

### THP-1 culture

THP-1 cells were obtained from ATCC (Lot # 62454382) and grown for multiple passages in T-75 flasks between 2-8 × 10^5^ cells/mL in growth medium containing RPMI-1640 (Corning), 10% fetal bovine serum, and 1% penicillin–streptomycin. For differentiation of THP-1 cells, non-adherent cells were diluted to 2 × 10^5^ cells/mL and grown overnight, and a final concentration of 100 nM PMA was added the following morning. THP-1 derived macrophages were collected after 72-h exposure to PMA by aspirating media and any non-adherent cells, and incubating adherent cells with TrypLE (ThermoFisher) for 15 min followed by cell wash in phosphate-buffered saline (PBS) buffer. Drug-disruption of POLR3G/Pol III was performed in THP-1 cells by treatment with RNA polymerase III inhibitor ML-60218 (Catalog# 557403-10MG, SigmaAldrich) at a final concentration of 25 μM and cells collected at 1, 2, 3, and 4 h post-exposure followed by cell wash in PBS buffer.

#### Small interfering RNA (siRNA) and Antisense Oligonucleotide (ASO) transfection

Knockdown of MYC was performed in THP-1 monocytes in media without penicillin–streptomycin, using Silencer Select predesigned siRNAs (Catalog# 439240, Invitrogen, ID s9129), mixed with OPTI-MEM media and Lipofectamine RNAiMAX Transfection Reagent (Catalog# 13778030, Invitrogen) per manufacturer’s instruction and added to THP-1 at a final concentration of 500 nM. A second knockdown step was carried out 48-h post transfection, and THP-1 lysate, total RNA, and small RNA were extracted on day 4 (96-h) using the mirVana PARIS RNA and Native Protein Purification kit (Catalog# AM1556, Invitrogen) following standard protocols. Custom Antisense Oligonucleotides were designed against SNAR-A core region (ASO #1, 5'-mG*-mC*-mC*-mU*-mC*-C*-G*-T*-G*-A*-G*-G*-G*-mC*-mC*-mC*-mG*-mA-3'), the SNAR-A tail region (ASO #2, 5'-mU*-mC*-mC*-mU*-mU*-T*-T*-T*-T*-C*-C*-G*-A*-C*-C*-mC*-mA*-mU*-mG*-mU-3'), and scramble control sequence (scramble ASO, 5'-mG*-mG*-mU*-mA*-mG*-G*-A*-G*-G*-T*-G*-C*-G*-T*-C*-mU*-mG*-mU*-mU*-mU-3') *m* = 2' O-Methyl RNA base; * = Phosphorothioate Bond (Integrated DNA Technologies). THP-1 cells were transfected with ASOs mixed with OPTI-MEM media and Lipofectamine RNAiMAX Transfection Reagent (Catalog# 13778030, Invitrogen) and added to THP-1 cells absent antibiotic selection at a final concentration of 1 uM. Small RNA was extracted 48-h post transfection using the mirVana PARIS RNA and Native Protein Purification kit (Catalog# AM1556, Invitrogen) following standard protocols.

### Lentiviral transduction

THP-1 monocytes were plated at 2 × 10^5^ cells/mL in 12-well plates overnight. A final concentration of 1 uM PMA was added the following morning, and THP-1 cells were allowed to differentiate for 72-h. Non-adherent cells were aspirated following 72-h exposure and replaced with fresh media containing 10 ug/mL polybrene (Catalog# sc-134220A, Santa Cruz Biotechnology). THP-1 macrophages were transduced with prepackaged lentiviral particles of either [1] copGFP control (Catalog# sc-108084, Santa Cruz Biotechnology), [2] control activation (Catalog# sc-437282, Santa Cruz Biotechnology), or [3] POLR3G activation (Catalog# sc-417072-LAC-2, Santa Cruz Biotechnology). THP-1 macrophages were spun in the presence of 200 uL activation particles / well at 300 × g for 45 min. Polybrene-containing media was replaced with fresh media 8-h post transduction. Confirmation of positive lentiviral transduction of THP-1 macrophages was monitored by GFP visualization and THP-1 macrophages harvested 72-h following transduction. Total and small RNA fractions were purified using the mirVana PARIS RNA and Native Protein Purification kit (Catalog# AM1556, Invitrogen) following standard protocols.

### Real-time quantitative PCR

mRNA changes in THP-1 monocyte and THP-1 macrophages were quantified using the Cells-to-CT 1-Step Taqman Kit (Catalog# A25603, Ambion), with the exception that total and small RNA fractions were instead extracted with the mirVana PARIS RNA and Native Protein Purification kit (Catalog# AM1556, Invitrogen), following standard protocols for separate isolation of total and small RNA. Real-time Quantitative PCR was performed with a QuantStudio 6 Flex Real-Time PCR System (Applied Biosystems) using TaqMan 1-Step qRT-PCR Mix and predesigned TaqMan Gene Expression Assays (20X; Catalog# 4331182 ThermoFisher) for selected genes: Hs02786624_g1 (GAPDH); Hs99999903_m1(ACTB); Hs04978644_g1 (POLR3G); Hs01113209_g1 (POLR3GL); Hs00153408_m1(MYC). Changes in small RNA were similarly profiled using the TaqMan MicroRNA Cells-to-CT Kit (Catalog# 4391848, Invitrogen) with the exception that small RNA fractions were instead extracted with the mirVana PARIS RNA and Native Protein Purification kit (Catalog# AM1556, Invitrogen), following standard protocol for separate isolation of total and small RNA. For small RNA, Reverse Transcription (RT) of purified small RNA was performed with a multiplex RT reaction step using an undiluted primer pool consisting of RNU19 (RT: 001003; PN4427975), Z30 (RT: 001092; PN4427975), SNAR-A context sequence 1 (RT: CTWCWVA; PN4398987 and RT: CTXGRE7; PN4398987), SNAR-A context sequence 2 (RT: CTYMJY4, PN4398987). Small RNA changes were measured using RT product via Real-time Quantitative PCR (QuantStudio 6 Flex Real-Time PCR System—Applied Biosystems) using TaqMan Master Mix (2×) and predesigned TaqMan MicroRNA Gene Expression Assays (20X; Catalog# 4331182 ThermoFisher) for selected genes: RNU19 (TM: 001003; PN4427975), Z30 (TM: 001092; PN4427975), SNAR-A (context sequence 1) (TM: CTWCWVA; PN4398987 and TM: CTXGRE7; PN4398987), SNAR-A (context sequence 2) (TM: CTYMJY4, PN4398987). SNAR-A context sequence 1 and context sequence 2 were purchased from ThermoFisher Scientific using the Custom TaqMan Small RNA Assay (Catalog# 4398987, Applied Biosystems) by submitting sequences corresponding to SNAR-A1 and SNAR-A13 (context sequence 1) and SNAR-A4 (context sequence 2). Context sequences provided by ThermoFisher Scientific: SNAR-A context sequence 1 = CCAGGGCACGAGUUCGAGGCCAGCCUGGUCCACAUGGGUCGGAAAAAAGGACUUUUUUUU; SNAR-A context sequence 2 = UCCAGGGCACGAGUUCGAGGCCAGCCUGGUCCACAUGGGUCGGAAAAAAGGAUUUUUUUU. mRNA and small RNA abundances presented were determined as the relative fold change normalized with two reference genes per RNA type (mRNA: *GAPDH* + *ACTB*, small RNA: *RNU19* + *Z30*).

### Cell viability and proliferation assay

THP-1 viability and proliferation were analyzed using the CyQUANT MTT Cell Viability Assay (Catalog# V13154, Invitrogen). 12 mM MTT was prepared as instructed by product protocol, adding 1 mL of PBS into 1 vial of 5 mg MTT. Vial was vortexed until completely dissolved and stored at 4 °C, protected from light. In a transparent 96-well tissue culture plate, 10 uL of 12 mM MTT was aliquoted into each well per data point, with an extra well of MTT for negative control. 100 uL of cell culture suspension was then added to the aliquoted MTT and gently pipetted up and down to mix. Cell culture suspension was well agitated before collecting sample to ensure homogenous suspension sampling. In negative control well, 100 uL of cell culture media was added. 96-well plate was then incubated at 37 °C, 5% CO2, for 4 h, protected from light. After incubation, plate was spun down for 5 min at 300 g. 85 uL of cell culture suspension was carefully removed from each well, without disturbing the sediment at the bottom. 50 uL of DMSO was then added and carefully pipetted up and down to mix, avoiding air bubbles. 96-well plate was then incubated for 5 min in 37 °C and then scanned using an M1000 Infinite Tecan Microplate reader. Absorbance was determined at 540 nm. Assay was repeated across 5 days and data plotted across time.

### Total, nuclear, cytoplasmic, and exosomal small RNA purification

Total steady-state small RNA was purified from equal numbers (~2 million cells per small RNA-seq experiment) of THP-1 monocytes ± ML-60218 exposure and THP-1 macrophages (72-h PMA) using the mirVana miRNA isolation kit according to instructions (Catalog# AM1560, Invitrogen). Nuclear and cytoplasmic THP-1 fractions were isolated using the NE-PER Nuclear and Cytoplasmic Extraction Reagents (Catalog# 78833, Thermo Scientific) according to instructions, with two additional nuclear washes prior to nuclear extraction. Fractionation purities were assessed by β-tubulin and Lamin B2 contamination (Supplementary Fig. 2i). THP-1 monocyte and THP-1 macrophage exosomes were isolated by differential centrifugation (10 min × 300 g, 10 min × 2000 g, 30 min × 10,000 g, 70 min × 100,000 g all at 4 °C), washed and resuspended in PBS for analysis. Exosome particle size distributions were assessed by nanoparticle tracking analysis (NanoSight) (Supplementary Fig. 2g). Small RNA was immediately isolated from subcellular fractionation and exosomal purification experiments using the mirVana miRNA isolation kit according to instructions. Small RNA libraries were generated using the NEBnext small RNA library preparation kit according to instructions (E7580S, New England BioLabs).

### Immunoblotting

THP-1 lysates were extracted using the mirVana PARIS RNA and Native Protein Purification kit (Catalog# AM1556, Invitrogen) following standard protocols, in tandem with total and small RNA isolation experiments where applicable. Total protein concentration was determined using Pierce BCA protein assay kit (Catalog# 23225, Thermo Scientific), and equivalent protein fractions, diluted in cell lysis buffer, were prepared in 4× NuPAGE LDS sample buffer (Catalog# NP0007, Invitrogen) and 10× NuPAGE reducing agent (Catalog# NP0004, Invitrogen). Proteins were separated on NuPAGE 4-12% Bis-Tris Protein Gels (Catalog# NP0329BOX, Invitrogen) using MES SDS Running Buffer (Catalog# NP000202, Invitrogen) and transferred onto polyvinylidene difluoride membrane 0.2 um (Catalog# LC2002, Invitrogen). Transfer membranes were blocked with 5% blotting-grade blocker, nonfat milk (Catalog# 1706404, BioRad), followed by incubation with primary antibody at 4^°^C overnight. Membranes were washed with TBST and incubated with secondary antibody conjugated with horseradish peroxidase (Catalog# 31462, Invitrogen) for 1 h at room temperature followed by addition wash in TBST. Proteins were visualized using either SuperSignal West Pico (Catalog# 34580, Thermo Scientific) or SuperSignal West Femto (Catalog# 34096, Thermo Scientific). Antibodies used for immunoblotting include Rabbit Anti-POLR3A (Abcam, ab96328 lot#GR318563) [1:1,000], Rabbit Anti-POLR3B (Bethyl, A301-855A) [1:1,000], Rabbit Anti-POLR3C (Bethyl, A303-063A) [1:1,000], Rabbit Anti-POLR3D (Bethyl, A302-295A) [1:1,000], Rabbit Anti-POLR3E (Bethyl, A303-708A) [1:333], Rabbit Anti-POLR3G (Invitrogen, PA5-51120 lot#UG2803044) [1:2,500], Rabbit Anti-POLR3GL (Novus Biologicals, NBP1-79826) [1:150], Rabbit Anti-MYC (Novus Biologicals, NBP2-43691) [1:1,000], Rabbit Anti-Lamin B2 (Cell Signaling Technologies, E1S1Q lot 1) [1:1,000], and Rabbit Anti-TUBB (Abcam, ab21058, lot# GR3280069-1) [1:5,000]. Source Data file includes uncropped immunoblot sections.

### Chromatin immunoprecipitation (ChIP)

Equal numbers of THP-1 monocytes, THP-1 derived macrophages, and ML-60218 treated monocytes were collected (~10 million cells per ChIP experiment) and resuspended in growth media at 1 × 10^6^ cells/mL and cross-linked with rotation at room temperature in 1% formaldehyde for 10 min. Cross-linking was quenched with the addition of 200 mM glycine and an additional 5 min of rotation at room temperature. Cross-linked cells were then spun down and resuspended in 1× RIPA lysis buffer, followed by chromatin shearing via sonication (3 cycles using a Branson sonicator: 30 s on, 60 s off; 20 additional cycles on a Bioruptor sonicator: 30 s on, 30 s off). Individual ChIP experiments were performed on pre-cleared chromatin using antibody-coupled ChIP grade Protein G magnetic beads (Cell Signaling Technology). POLR3A antibody was obtained from Abcam (ab96328 lot#GR318563). POLR3B antibody was obtained from Bethyl (A301-855A). POLR1D antibody was obtained from Bethyl (A304-847A). POLR3C antibody was obtained from Bethyl (A303-063A). POLR3G antibody was obtained from Invitrogen (PA5-51120 lot#UG2803044). POLR3GL antibody was obtained from Novus Biologicals (NBP1-79826). POLR3D antibody was obtained from Bethyl (A302-295A). POLR3E antibody was obtained from Bethyl (A303-708A). BRF1 antibody was obtained from Abcam (ab74221). GTF3C1 antibody was obtained from SigmaAldrich (PLA0180). IgG antibody was obtained from ThermoFisher Scientific (NeoMarkers NC-100-P0). 5 ug of antibody per ChIP was coupled to 18 uL of beads and rotated overnight with sheared chromatin at 4°C. Beads were then washed 5× in ChIP wash buffer (Santa Cruz), 1× in TE, and chromatin eluted in TE + 1% SDS. Cross-linking was then reversed by incubation at 65°C overnight, followed by digestion of RNA (30 min RNase incubation at 37°C) and digestion of protein (30 min proteinase K incubation at 45°C). ChIP DNA was then purified on a minElute column (Qiagen), followed by DNA library preparation (NEBNext Ultra II DNA Library Prep Kit for Illumina) and size selection of 350-550 bp fragments via gel extraction (Qiagen).

### Data acquisition

Previously reported THP-1 RNA-seq, ATAC-seq, and nascent small RNA sequencing files were obtained from Gene Expression Omnibus (GEO) series GSE96800. ATAC-seq and gene expression data derived from primary immune progenitor and primary differentiated immune cell populations were obtained from GEO series GSE74912 and GSE118119, respectively. NIH Roadmap Epigenomics gene expression profiles related to immortalized cell line (ENCODE) and multi-tissue Pol III subunit levels (Supplementary Fig. 2a,b) were extracted from the “57epigenomes.RPKM.pc” file obtained from: https://egg2.wustl.edu/roadmap/web_portal/ /processed_data.html#RNAseq_uni_proc. The Cancer Genome Atlas (TCGA) primary solid tumor Pol III subunit expression levels were extracted across all disease cohorts from the Broad Institute TCGA Genome Data Analysis Center (GDAC) Firehose mRNASeq Level 3 RSEM gene normalized data files at https://gdac.broadinstitute.org. Primary solid tumor ATAC-seq alignment bam files were retrieved from the Genomic Data Commons Data Portal (https://portal.gdc.cancer.gov). Clinical patient survival outcomes and Kaplan–Meier survival probability data were retrieved from the Kaplan–Meier Plotter Pan-cancer RNA-seq analysis platform (https://kmplot.com/analysis/index.php?p=service&cancer=pancancer_rnaseq)^92^. High POLR3G, POLR3GL, or MYC expression were defined using a uniform upper tertile patient split across all cancer types, compared to the remaining cohort as shown. KMplot data visuals were generated using R packages surviplot (https://github.com/aroneklund/surviplot) and survival (https://github.com/therneau/survival). Model cell-type illustrations were created using BioRender.com.

### ChIP-seq analysis

For each individual factor-specific ChIP-seq experiment, biological replicates and experimental conditions (-PMA/ML-60218; +PMA; + ML-60218) were sequenced together on an Illumina HiSeq4000 (paired-end, 100 bp). Sequencing was performed by the Genome Sequencing Service Center by Stanford Center for Genomics and Personalized Medicine. Sequencing reads were trimmed using Trim Galore v.0.4.0 (https://github.com/FelixKrueger/TrimGalore) prior to downstream sequence alignment and analyses. Trimmed paired-end ChIP sequencing reads were mapped to the GRCh38 genome using bowtie version 2.2.4 with settings “bowtie2 -t –sensitive -x”^93^ and processed with samtools version 1.3.1^94^. Read counts were extracted over a comprehensive RNAcentral database annotation of noncoding RNAs^55,56^ using bedtools version 2.23.0^95^. Gene annotation set and corresponding GRCh38 coordinates were downloaded from the contemporaneous RNAcentral database (release version13):ftp://ftp.ebi.ac.uk/pub/databases/RNAcentral/releases/13.0/genome_coordinates/bed/homo_sapiens.GRCh38.bed.gz. RNAcentral coordinate IDs were mapped to Ensembl, Gencode, GtRNAdb, HGNC, Lncbase, Lncbook, Lncipedia, Lndcrnadb, miRbase, NONCODE, Refseq, and Rfam. To remove duplicate coordinate gene entries derived from multiple databases, genes with start and end coordinates within 50 bp of each other were merged into singular coordinate entries. Gene-specific raw ChIP-seq signal counts were extracted and include the flanking 150 bp for each RNAcentral gene coordinate entry. ChIP-seq data were used to compare Pol III subunit binding patterns (e.g., Pol III subunit map: Supplementary Fig. 1a) and intra-subunit dynamics (e.g., dynamic POLR3G occupancy during THP-1 differentiation: Fig. 2c). Inter-subunit and intra-subunit comparisons were analyzed differently. For cross-comparison of Pol III subunit occupancy (inter-subunit), ChIP-seq counts corresponding to each individual Pol III subunit experiment were, together, rank normalized over a subset of canonical Pol III-transcribed genes annotated in the RNAcentral database, including tRNA, 5 S rRNA, U6, 7SK, 7SL, SNAR, Y, vault, RPPH1, RMRP, BCYRN1. In total, 11,526 potential Pol III-transcribed genes, including numerous pseudogenes, were allowed for initial analysis of Pol III occupancy. Rank normalization seeks to account for signal-to-noise variation across all subunit-specific chromatin IP experiments. After ranked assessment of Pol III subunit co-localization (Supplementary Fig. 1a), 350 unique Pol III-transcribed genes were considered confidently bound by the Pol III complex and likely to be transcriptionally active and thus included in downstream integrated analyses with small RNA abundance. For statistical analysis of individual Pol III subunit changes (intra-subunit), differential count statistics corresponding to Pol III subunit occupancy changes were derived from the exactTest function of the edgeR package for differential expression analysis^96,97^ over raw counts extracted over the entire RNAcentral annotation set. All downstream visualization and analyses were then subset on the 350 canonical Pol III-transcribed genes identified as described (Supplementary Fig. 1a). ChIP-seq signal track data were generated from post-filtering read alignment bam files using the deeptools bamCompare tool^98,99^. Signal track visualization plots were generated using UCSC tools version 3.0.9^100^ and the Sushi package for genomic visualization^101^.

### Small RNA-seq analysis

For analysis of Pol III-transcribed small RNA abundance, we mapped nascent and steady-state small RNA reads to the entire genome space (GRCh38) to avoid false-positive signal arising from sequence reads unrelated to Pol III-transcribed genes that might otherwise occur using a limited reference set^102^. Sequencing reads were trimmed using trim galore v.0.4.0 prior to downstream sequence alignment and analyses. By default, multi-mapping reads were reported as a singular best alignment. Read counts were extracted over a comprehensive RNAcentral database annotation of noncoding RNAs^55,56^. Gene annotation set and corresponding GRCh38 coordinates were downloaded from the contemporaneous RNAcentral database (release version13): ftp://ftp.ebi.ac.uk/pub/databases/RNAcentral/releases/13.0/genome_coordinates/bed/homo_sapiens.GRCh38.bed.gz. RNAcentral coordinate IDs were mapped to Ensembl, Gencode, GtRNAdb, HGNC, Lncbase, Lncbook, Lncipedia, Lndcrnadb, miRbase, noncode, Refseq, and Rfam. To remove duplicate coordinate gene entries derived from multiple databases, genes with start and end coordinates within 50 bp of each other were merged into singular coordinate entries. Gene-specific raw small RNA signal counts were extracted and include the flanking 25 bp for each RNAcentral gene coordinate entry. Differential count statistics corresponding to small RNA changes were derived from the exactTest function of the edgeR package for differential expression analysis^96,97^ over the entire RNAcentral annotation set. Downstream analyses and visualizations subset on canonical Pol III-transcribed genes and, specifically, the 350 genes confidently identified as Pol III-occupied in our system by ChIP-seq analysis.

### Correlation between Pol III subunit expression and gene chromatin features

[A] *Primary Immune cell populations*: Simultaneous ATAC-seq and gene expression data derived from primary immune progenitor and primary differentiated immune cell populations were obtained from GEO series GSE74246, GSE74912, GSE118165, and GSE118119^64,65^. Pol III subunit expression profiles were extracted from previously processed gene abundance analysis files (GSE74246_RNAseq_All_counts.txt and GSE118165_RNA_gene _abundance.txt). For the purpose of Pol III identity and gene accessibility correlations, the log2 ratio of POLR3G and POLR3GL abundance levels within individual samples was considered. Raw ATAC-seq data were downloaded and mapped to the GRCh38 genome using bowtie version 2.2.4 with settings “bowtie2 -t –sensitive -x”^93^. Read counts were extracted over a comprehensive RNAcentral database annotation of noncoding RNAs^55,56^. Gene annotation set and corresponding GRCh38 coordinates were downloaded from the contemporaneous RNAcentral database (release version13): ftp://ftp.ebi.ac.uk/pub/databases/RNAcentral/releases/13.0/genome_coordinates/bed/homo_sapiens.GRCh38.bed.gz. Gene-specific raw ATAC-seq signal counts were extracted and include the flanking 150 bp for each RNAcentral gene coordinate entry. Gene accessibility signal counts were rank normalized across 248 ATAC-seq experiments, including unstimulated and stimulated immune cell experiments^65^. In total, 188 simultaneous ATAC-seq and RNA-seq profiles were matched by sample ID, and the Pearson correlation determined for the log2(POLR3G/POLR3GL) ratio and normalized gene accessibility profile in individual samples (Supplementary Fig. 3r). Analogous Pol III identity and gene chromatin feature correlation analysis was performed against 10 aggregate cell populations, including progenitor, myeloid, B cells (unstimulated), natural killer (NK) cells (unstimulated), CD4 + T cells (unstimulated), CD8 + T cells (unstimulated), and γδ T cells (unstimulated), pre-leukemic hematopoietic stem cells (pHSCs), leukemia stem cells (LSC), and leukemic blast cells (Blast) (Supplementary Fig. 3f).

[B] *The Cancer Genome Atlas* (TCGA): Pol III subunit expression levels were extracted across all TCGA disease cohorts from the Broad Institute TCGA Genome Data Analysis Center (GDAC) Firehose mRNASeq Level 3 RSEM gene normalized data files at https://gdac.broadinstitute.org. For analysis of Pol III subunit expression changes in individual disease cohorts, patient-matched normal tissue (11) and primary solid tumor (01) expression profiles were merged by participant ID. Disease cohorts with ≥5 patient-matched samples were considered (BLCA, *n* = 19; BRCA, *n* = 115; CHOL, *n* = 9; COADREAD, *n* = 32; ESCA, *n* = 11; KICH, *n* = 25; LUAD, *n* = 58; LUSC, *n* = 51; PRAD, *n* = 52; STAD, *n* = 32; THCA, *n* = 59; UCEC, *n* = 10). TCGA POLR3G/POLR3GL expression ratios were derived as the intra-sample ratio of RSEM gene normalized POLR3G expression counts/POLR3GL expression counts, an internally controlled comparative measure of the relationship between POLR3G and POLR3GL in either normal or tumor samples. Primary solid tumor ATAC-seq alignment bam files were retrieved from the Genomic Data Commons Data Portal (https://portal.gdc.cancer.gov). Read counts were extracted over a comprehensive RNAcentral database annotation of noncoding RNAs^55,56^. Gene annotation set and corresponding GRCh38 coordinates were downloaded from the contemporaneous RNAcentral database (release version13): ftp://ftp.ebi.ac.uk/pub/databases/RNAcentral/releases/13.0/genome_coordinates/bed/ homo_sapiens.GRCh38.bed.gz. Gene-specific raw ATAC-seq signal counts were extracted and include the flanking 150 bp for each RNAcentral gene coordinate entry. Gene accessibility signal counts were rank normalized across 410 TCGA ATAC-seq experiments. In total, 388 ATAC-seq and RNA-seq profiles were matched by primary solid sample ID, and the Pearson correlation determined for the log2(POLR3G/POLR3GL) ratio and normalized gene accessibility profile in individual samples (Supplementary Fig. 4e).

### Multi-experiment overlapping gene enrichment analysis

Moving overlap analysis identifies the number of genes, when ranked by degree of gene loss (e.g., Pol III occupancy, small RNA abundance ± PMA; ML-60218), that are shared between 2 or more independent experiments (e.g., dynamic Pol III occupancy v. dynamic small RNA abundance PMA; PMA-sensitive v. ML-60218 sensitive RNA abundance, etc.) at a given cutoff level wherein the number of top genes (*n*) are considered. Maximum enrichment scores (Fig. 5k) represent the highest observed/expected ratio for a given gene based on representation within an overlapping gene set along the moving plot.

### Reporting summary

Further information on research design is available in the Nature Research Reporting Summary linked to this article.

## Supplementary information

Supplementary Information

Reporting Summary

## Data Availability

The data supporting the findings of this study are available from the corresponding authors upon reasonable request. The ChIP and small RNA sequencing data generated in this study have been deposited through the NCBI Gene Expression Omnibus under GEO series number GSE163422 and series number GSE171884, and are publicly available. Source data for the figures and supplementary figures are provided as a Source Data file. Source data are provided with this paper.

## Source data

Source Data

## References

[1] Geiduschek EP, Kassavetis GA (2001). The RNA polymerase III transcription apparatus. J. Mol. Biol..

[2] Nikitina TV, Tishchenko LI (2005). [RNA polymerase III transcription apparatus: structure and transcription regulation]. Mol. Biol. (Mosk.).

[3] Canella D, Praz V, Reina JH, Cousin P, Hernandez N (2010). Defining the RNA polymerase III transcriptome: Genome-wide localization of the RNA polymerase III transcription machinery in human cells. Genome Res..

[4] James Faresse N (2012). Genomic study of RNA polymerase II and III SNAPc-bound promoters reveals a gene transcribed by both enzymes and a broad use of common activators. PLoS Genet..

[5] Dieci G, Conti A, Pagano A, Carnevali D (2013). Identification of RNA polymerase III-transcribed genes in eukaryotic genomes. Biochim Biophys. Acta.

[6] Peterlin BM, Brogie JE, Price DH (2012). 7SK snRNA: a noncoding RNA that plays a major role in regulating eukaryotic transcription. Wiley Interdiscip. Rev. RNA.

[7] Egloff S, Studniarek C, Kiss T (2018). 7SK small nuclear RNA, a multifunctional transcriptional regulatory RNA with gene-specific features. Transcription.

[8] Didychuk AL, Butcher SE, Brow DA (2018). The life of U6 small nuclear RNA, from cradle to grave. RNA.

[9] Bohnsack MT, Sloan KE (2018). Modifications in small nuclear RNAs and their roles in spliceosome assembly and function. Biol. Chem..

[10] Goldfarb KC, Cech TR (2017). Targeted CRISPR disruption reveals a role for RNase MRP RNA in human preribosomal RNA processing. Genes Dev..

[11] Thiel CT (2005). Severely incapacitating mutations in patients with extreme short stature identify RNA-processing endoribonuclease RMRP as an essential cell growth regulator. Am. J. Hum. Genet.

[12] Baer M, Nilsen TW, Costigan C, Altman S (1990). Structure and transcription of a human gene for H1 RNA, the RNA component of human RNase P. Nucleic Acids Res..

[13] Jarrous N (2017). Roles of RNase P and its subunits. Trends Genet..

[14] Walter P, Blobel G (1982). Signal recognition particle contains a 7S RNA essential for protein translocation across the endoplasmic reticulum. Nature.

[15] Doudna JA, Batey RT (2004). Structural insights into the signal recognition particle. Annu Rev. Biochem..

[16] Hahne J. C., Lampis A., Valeri N. Vault RNAs: hidden gems in RNA and protein regulation. *Cell Mol. Life Sci*. 10.1007/s00018-020-03675-9 (2020). 10.1007/s00018-020-03675-9 PMC7904556 33063126

[17] Horos R (2019). The small non-coding vault RNA1-1 acts as a riboregulator of autophagy. Cell.

[18] Kowalski MP, Krude T (2015). Functional roles of non-coding Y RNAs. Int J. Biochem Cell Biol..

[19] Tebaldi T (2018). HuD is a neural translation enhancer acting on mTORC1-responsive genes and counteracted by the Y3 small non-coding RNA. Mol. Cell.

[20] Tiedge H, Chen W, Brosius J (1993). Primary structure, neural-specific expression, and dendritic location of human BC200 RNA. J. Neurosci..

[21] Parrott AM (2011). The evolution and expression of the snaR family of small non-coding RNAs. Nucleic Acids Res..

[22] Parrott AM, Mathews MB (2007). Novel rapidly evolving hominid RNAs bind nuclear factor 90 and display tissue-restricted distribution. Nucleic Acids Res..

[23] Samson J, Cronin S, Dean K (2018). BC200 (BCYRN1) - The shortest, long, non-coding RNA associated with cancer. Noncoding RNA Res..

[24] Booy EP, McRae EK, Koul A, Lin F, McKenna SA (2017). The long non-coding RNA BC200 (BCYRN1) is critical for cancer cell survival and proliferation. Mol. Cancer.

[25] Shi Z (2019). Long non-coding RNA snaR is involved in the metastasis of liver cancer possibly through TGF-β1. Oncol. Lett..

[26] Huang Y, Hu Y, Jin Z, Shen Z (2018). LncRNA snaR upregulates GRB2-associated binding protein 2 and promotes proliferation of ovarian carcinoma cells. Biochem Biophys. Res. Commun..

[27] Lee J (2017). Biological function of long noncoding RNA snaR in HER2-positive breast cancer cells. Tumour Biol..

[28] Schramm L, Hernandez N (2002). Recruitment of RNA polymerase III to its target promoters. Genes Dev..

[29] Grewal SS (2015). Why should cancer biologists care about tRNAs? tRNA synthesis, mRNA translation and the control of growth. Biochim Biophys. Acta.

[30] Chen CY (2018). Maf1 and repression of RNA polymerase III-mediated transcription drive adipocyte differentiation. Cell Rep..

[31] Vannini A (2010). Molecular basis of RNA polymerase III transcription repression by Maf1. Cell.

[32] Orioli A, Praz V, Lhote P, Hernandez N (2016). Human MAF1 targets and represses active RNA polymerase III genes by preventing recruitment rather than inducing long-term transcriptional arrest. Genome Res.

[33] Willis IM (2018). Maf1 phenotypes and cell physiology. Biochim Biophys. Acta Gene Regul. Mech..

[34] Willis IM, Moir RD (2018). Signaling to and from the RNA polymerase III transcription and processing machinery. Annu Rev. Biochem.

[35] Bonhoure N (2020). MAF1 is a chronic repressor of RNA polymerase III transcription in the mouse. Sci. Rep..

[36] Moqtaderi Z (2010). Genomic binding profiles of functionally distinct RNA polymerase III transcription complexes in human cells. Nat. Struct. Mol. Biol..

[37] Raha D (2010). Close association of RNA polymerase II and many transcription factors with Pol III genes. Proc. Natl Acad. Sci. USA.

[38] Oler AJ (2010). Human RNA polymerase III transcriptomes and relationships to Pol II promoter chromatin and enhancer-binding factors. Nat. Struct. Mol. Biol..

[39] Barski A (2010). Pol II and its associated epigenetic marks are present at Pol III-transcribed noncoding RNA genes. Nat. Struct. Mol. Biol..

[40] Alla RK, Cairns BR (2014). RNA polymerase III transcriptomes in human embryonic stem cells and induced pluripotent stem cells, and relationships with pluripotency transcription factors. PLoS One.

[41] Van Bortle K, Phanstiel DH, Snyder MP (2017). Topological organization and dynamic regulation of human tRNA genes during macrophage differentiation. Genome Biol..

[42] Ramsay EP (2020). Structure of human RNA polymerase III. Nat. Commun..

[43] Wang Z, Roeder RG (1997). Three human RNA polymerase III-specific subunits form a subcomplex with a selective function in specific transcription initiation. Genes Dev..

[44] Kassavetis GA, Geiduschek EP (2006). Transcription factor TFIIIB and transcription by RNA polymerase III. Biochem. Soc. Trans..

[45] Kenneth NS, Marshall L, White RJ (2008). Recruitment of RNA polymerase III in vivo. Nucleic Acids Res..

[46] Hoffmann NA (2015). Molecular structures of unbound and transcribing RNA polymerase III. Nature.

[47] Abascal-Palacios G, Ramsay EP, Beuron F, Morris E, Vannini A (2018). Structural basis of RNA polymerase III transcription initiation. Nature.

[48] Wong RC (2011). A novel role for an RNA polymerase III subunit POLR3G in regulating pluripotency in human embryonic stem cells. Stem Cells.

[49] Lund RJ (2017). RNA polymerase III subunit POLR3G regulates specific subsets of PolyA. Stem Cell Rep..

[50] Wang X, Gerber A, Chen WY, Roeder RG (2020). Functions of paralogous RNA polymerase III subunits POLR3G and POLR3GL in mouse development. Proc. Natl Acad. Sci. USA.

[51] Haurie V (2010). Two isoforms of human RNA polymerase III with specific functions in cell growth and transformation. Proc. Natl Acad. Sci. USA.

[52] Renaud M (2014). Gene duplication and neofunctionalization: POLR3G and POLR3GL. Genome Res..

[53] Wang Q (2021). Structural insights into transcriptional regulation of human RNA polymerase III. Nat. Struct. Mol. Biol..

[54] Girbig M (2021). Cryo-EM structures of human RNA polymerase III in its unbound and transcribing states. Nat. Struct. Mol. Biol..

[55] The RNAcentral Consortium. (2019). RNAcentral: a hub of information for non-coding RNA sequences. Nucleic Acids Res..

[56] Petrov AI (2015). RNAcentral: an international database of ncRNA sequences. Nucleic Acids Res..

[57] Chanput W, Mes JJ, Wichers HJ (2014). THP-1 cell line: an in vitro cell model for immune modulation approach. Int. Immunopharmacol..

[58] Kouno T (2013). Temporal dynamics and transcriptional control using single-cell gene expression analysis. Genome Biol..

[59] Tsuchiya S (1982). Induction of maturation in cultured human monocytic leukemia cells by a phorbol diester. Cancer Res..

[60] Daigneault M, Preston JA, Marriott HM, Whyte MK, Dockrell DH (2010). The identification of markers of macrophage differentiation in PMA-stimulated THP-1 cells and monocyte-derived macrophages. PLoS One.

[61] van Balkom BW, Eisele AS, Pegtel DM, Bervoets S, Verhaar MC (2015). Quantitative and qualitative analysis of small RNAs in human endothelial cells and exosomes provides insights into localized RNA processing, degradation and sorting. J. Extracell. Vesicles.

[62] Abramowicz A., Story M. D. The long and short of it: the emerging roles of non-coding RNA in small extracellular vesicles. *Cancers (Basel)*. 10.3390/cancers12061445 (2020). 10.3390/cancers12061445 PMC7352322 32498257

[63] Zhu L (2019). Exosomal tRNA-derived small RNA as a promising biomarker for cancer diagnosis. Mol. Cancer.

[64] Corces MR (2016). Lineage-specific and single-cell chromatin accessibility charts human hematopoiesis and leukemia evolution. Nat. Genet.

[65] Calderon D (2019). Landscape of stimulation-responsive chromatin across diverse human immune cells. Nat. Genet.

[66] Van Belle K (2016). Comparative in vitro immune stimulation analysis of primary human B cells and B cell lines. J. Immunol. Res.

[67] Trickett A, Kwan YL (2003). T cell stimulation and expansion using anti-CD3/CD28 beads. J. Immunol. Methods.

[68] Liu X (2020). Increased expression of POLR3G predicts poor prognosis in transitional cell carcinoma. PeerJ.

[69] Corces M. R. et al. The chromatin accessibility landscape of primary human cancers. *Science*. 10.1126/science.aav1898 (2018). 10.1126/science.aav1898 PMC6408149 30361341

[70] Hutter C, Zenklusen JC (2018). The cancer genome atlas: creating lasting value beyond Its Data. Cell.

[71] Wu L (2003). Novel small-molecule inhibitors of RNA polymerase III. Eukaryot. Cell.

[72] Kessler AC, Maraia RJ (2021). The nuclear and cytoplasmic activities of RNA polymerase III, and an evolving transcriptome for surveillance. Nucleic Acids Res.

[73] Petrie JL (2019). Effects on prostate cancer cells of targeting RNA polymerase III. Nucleic Acids Res.

[74] Zheng R (2019). Cistrome Data Browser: expanded datasets and new tools for gene regulatory analysis. Nucleic Acids Res.

[75] Mei S (2017). Cistrome Data Browser: a data portal for ChIP-Seq and chromatin accessibility data in human and mouse. Nucleic Acids Res.

[76] Liu T (2011). Cistrome: an integrative platform for transcriptional regulation studies. Genome Biol..

[77] Lin CY (2012). Transcriptional amplification in tumor cells with elevated c-Myc. Cell.

[78] Tesi A (2019). An early Myc-dependent transcriptional program orchestrates cell growth during B-cell activation. EMBO Rep..

[79] Marchingo J. M., Sinclair L. V., Howden A. J.,& Cantrell D. A. Quantitative analysis of how Myc controls T cell proteomes and metabolic pathways during T cell activation. *Elife*. 10.7554/eLife.53725 (2020). 10.7554/eLife.53725 PMC7056270 32022686

[80] Wang R (2011). The transcription factor Myc controls metabolic reprogramming upon T lymphocyte activation. Immunity.

[81] Dang CV (2012). MYC on the path to cancer. Cell.

[82] Topham C (2015). MYC is a major determinant of mitotic cell fate. Cancer Cell.

[83] Lee J (2016). Long noncoding RNA snaR regulates proliferation, migration and invasion of triple-negative breast cancer cells. Anticancer Res.

[84] Stribling D (2021). A noncanonical microRNA derived from the snaR-A noncoding RNA targets a metastasis inhibitor. RNA.

[85] Parrott AM, Walsh MR, Mathews MB (2007). Analysis of RNA:protein interactions in vivo: identification of RNA-binding partners of nuclear factor 90. Methods Enzymol..

[86] Li K (2020). ILF3 is a substrate of SPOP for regulating serine biosynthesis in colorectal cancer. Cell Res.

[87] Ghafouri-Fard S, Dashti S, Hussen BM, Farsi M, Taheri M (2021). BCYRN1: An oncogenic lncRNA in diverse cancers. Pathol. Res. Pr..

[88] Zhai H., Li Y. BCYRN1 is correlated with progression and prognosis in gastric cancer. *Biosci Rep*. 10.1042/BSR20190505 (2019). 10.1042/BSR20190505 PMC6859112 31652309

[89] Wang Y, Bai W, Wang M, Yu T, Zhang W (2019). Long non-coding RNA brain cytoplasmic RNA 1 acts as an oncogene and regulates cell proliferation and metastasis in non-small cell lung cancer. J. Nanosci. Nanotechnol..

[90] Hu T, Lu YR (2015). BCYRN1, a c-MYC-activated long non-coding RNA, regulates cell metastasis of non-small-cell lung cancer. Cancer Cell Int.

[91] Gerber A, Ito K, Chu CS, Roeder RG (2020). Gene-specific control of tRNA expression by RNA polymerase II. Mol. Cell.

[92] Nagy Á, Munkácsy G, Győrffy B (2021). Pancancer survival analysis of cancer hallmark genes. Sci. Rep..

[93] Langmead B, Trapnell C, Pop M, Salzberg SL (2009). Ultrafast and memory-efficient alignment of short DNA sequences to the human genome. Genome Biol..

[94] Li H (2009). The sequence alignment/Map format and SAMtools. Bioinformatics.

[95] Quinlan AR, Hall IM (2010). BEDTools: a flexible suite of utilities for comparing genomic features. Bioinformatics.

[96] Nikolayeva O, Robinson MD (2014). edgeR for differential RNA-seq and ChIP-seq analysis: an application to stem cell biology. Methods Mol. Biol..

[97] Robinson MD, McCarthy DJ, Smyth GK (2010). edgeR: a bioconductor package for differential expression analysis of digital gene expression data. Bioinformatics.

[98] Ramirez F (2016). deepTools2: a next generation web server for deep-sequencing data analysis. Nucleic Acids Res..

[99] Ramirez F, Dundar F, Diehl S, Gruning BA, Manke T (2014). deepTools: a flexible platform for exploring deep-sequencing data. Nucleic Acids Res.

[100] Kuhn RM, Haussler D, Kent WJ (2013). The UCSC genome browser and associated tools. Brief. Bioinform..

[101] Phanstiel DH, Boyle AP, Araya CL, Snyder MP (2014). Sushi.R: flexible, quantitative and integrative genomic visualizations for publication-quality multi-panel figures. Bioinformatics.

[102] Telonis AG, Loher P, Kirino Y, Rigoutsos I (2016). Consequential considerations when mapping tRNA fragments. BMC Bioinforma..

