# Supplementary material for: A cancer-associated RNA polymerase III identity drives robust transcription and expression of snaR-A noncoding RNA
Source: Nat Commun. 2022 May 30;13:3007. doi: 10.1038/s41467-022-30323-6 (PMC9151912; doi:10.1038/s41467-022-30323-6)
Supplement: Supplementary file 1 — Supplementary Information [file 41467_2022_30323_MOESM1_ESM.pdf]

Supplementary Information

A cancer-associated RNA polymerase III identity drives robust transcription and expression of snaR-A noncoding RNA

Kevin Van Bortle[1,2,3], David P. Marciano[1], Qing Liu[1,4,5], Tristan Chou[1], Andrew M. Lipchik[1,6], Sanjay Gollapudi[7], Benjamin S. Geller[1,8], Emma Monte[1], Rohinton T. Kamakaka[8], Michael P. Snyder[1*]

[1]Department of Genetics, Stanford University, Stanford, CA 94305, USA.

[2]Department of Cell & Developmental Biology, University of Illinois Urbana-Champaign, Urbana, IL 61801, USA

[3]Cancer Center at Illinois, University of Illinois Urbana-Champaign, Urbana, IL 61801, USA

[4]Stanford Cardiovascular Institute, Stanford University School of Medicine, Stanford, CA 94305, USA.

[5]Department of Physiology, Southern Illinois University School of Medicine, Carbondale, IL 62901, USA

[6]Department of Pharmaceutical Sciences, Eugene Applebaum College of Pharmacy and Health Sciences, Wayne State University, Detroit, MI 48201, USA

[7]Genomics Research Internship Program at Stanford, Stanford University, Stanford, CA 94305, USA.

[8]Department of Molecular, Cell, and Developmental Biology, University of Santa Cruz, Santa Cruz, CA 95064, USA.

* Correspondence to MPS (mpsnyder@stanford.edu)

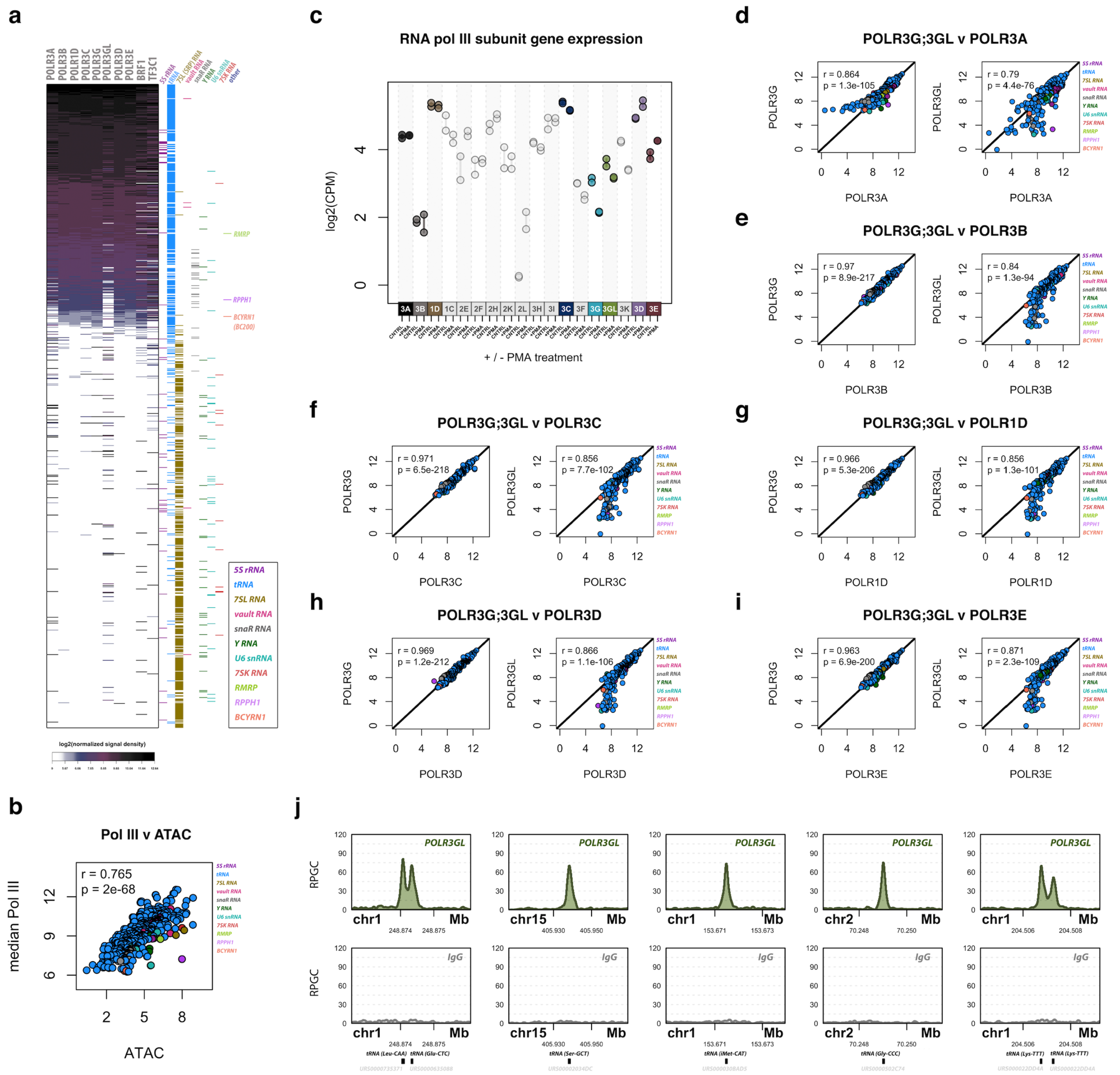

Supplementary Figure 1 I Pol III subunit expression, genomic occupancy, and chromatin accessibility. (**a**) Heatmap visualization of ChIP-seq signal density over individual Pol III-transcribed genes for Pol III subunits POLR3A, POLR3B, POLR1D, POLR3C, POLR3G, POLR3GL, POLR3D, and POLR3E, TFIIIB subunit BRF1, and TFIIIC subunit TF3C1. Heatmap is ordered by the median signal density across Pol III subunits over canonical Pol III-transcribed genes (n = top 1,000 canonical genes; RNAcentral annotation). Corresponding gene type indicated by right-flanking colorbar. (**b**) Correlation between chromatin accessibility profile (ATAC-seq) and median Pol III subunit signal enrichment. Pearson's correlation, *p*-value computed as a two-sided test. (**c**) Gene expression profile for Pol III subunits in THP-1 monocytes as well as PMA-treated THP-1 macrophages (points represent individual biological replicates, mapped subunits are colored). (**d-i**) Correlation scores between either POLR3G or POLR3GL and mapped Pol III subunits POLR3A (**d**), POLR3B (**e**), POLR3C (**f**), POLR1D (**g**), POLR3D (h), and POLR3E (**i**). Pearson's correlation, *p*-value computed as a two-sided test. (**j**) Representative example ChIP-seq signal plots for POLR3GL (top) and IgG control experiments (bottom) at Pol III-occupied genes, confirming POLR3GL signal at canonical target sites. Source Data are provided as a Source Data file.

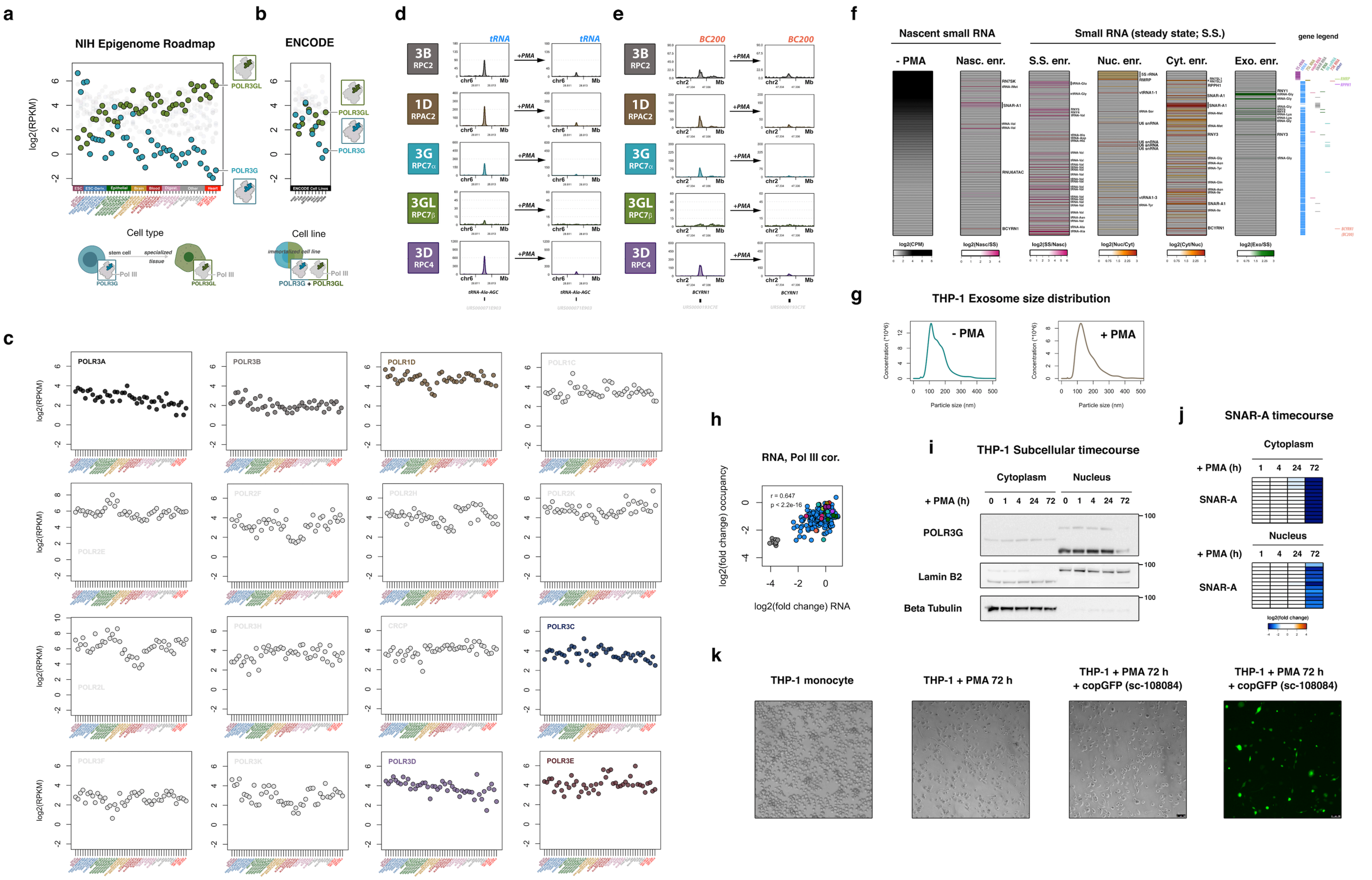

Supplementary Figure 2 | Dynamic Pol III subunit expression, occupancy, and compartmental small RNA abundance in THP-1. (**a**) Gene expression levels of mapped Pol III subunits across distinct cellular and tissue contexts with emphasis on *POLR3G* and *POLR3GL* gene expression in embryonic stem cells (ESCs), ESC-derived, epithelial, brain, blood, digestive, heart, and other contexts (NIH epigenome roadmap). (**b**) Gene expression levels of mapped Pol III subunits across immortalized ENCODE cell lines. (**c**) Analogous gene expression plots for all other subunits of the Pol III complex; mapped subunits are colored. (**d**) ChIP-seq profile for POLR3B, POLR1D, POLR3G, POLR3GL, and POLR3D are shown in THP-1 cells before (left) and after 72-hour PMA treatment (right) over tRNA gene *Ala-AGC* (URS000071E903). (**e**) Analogous ChIP-seq signal profile over *BCYRN1* gene encoding BC200 RNA before and after 72-hour PMA treatment in THP-1 cells. (**f**) Heatmap visualization of small RNA profiles in THP-1 monocytes, including nascent, steady-state, nuclear, cytoplasmic, and exosomal small RNA enrichment. All heatmaps are ordered by the level of nascent RNA abundance in THP-1 monocytes. Corresponding genes for all heatmaps indicated by color legend on right. (**g**) Purified THP-1 exosome nanoparticle size analysis. THP-1 monocyte and THP-1 macrophage exosomes were isolated by differential centrifugation and exosome particle size distributions were assessed by nanoparticle tracking analysis (NTA; NanoSight). Plots represent NTA of particle concentration (particle/mL) and size distribution of THP-1 monocyte exosomes (left) and THP-1 macrophage exosomes (right). (**h**) Correlation between the median Pol III subunit fold change and median change in RNA abundance in THP-1 cells +/- PMA. Pearson's correlation, *p*-value computed as a two-sided test (**i**) Subcellular fractionation time course immunoblot for POLR3G, Lamin B2 (nuclear marker), and Beta Tubulin (cytoplasm marker) protein levels in THP-1 cells at 0, 1, 4, 24, and 72-hour post PMA treatment. Immunoblot represents subcellular fractionation experiments corresponding to nuclear and cytoplasmic small RNA purification. Observations representative of 2 independent experiments. (j) Heatmap visualization of dynamic snaR-A ncRNA levels (log2 fold change) in nuclear and cytoplasmic small RNA fractions at 0, 1, 4, 24, and 72-hour post PMA treatment. (**k**) Imaging of THP-1 cells +/- PMA differentiation; positive lentiviral transduction of THP-1 macrophages was monitored using copGFP control lentiviral particles (sc-108084). Observations representative of 3 or more independent experiments. Molecular weights are indicated in kDa. Source Data are provided as a Source Data file.

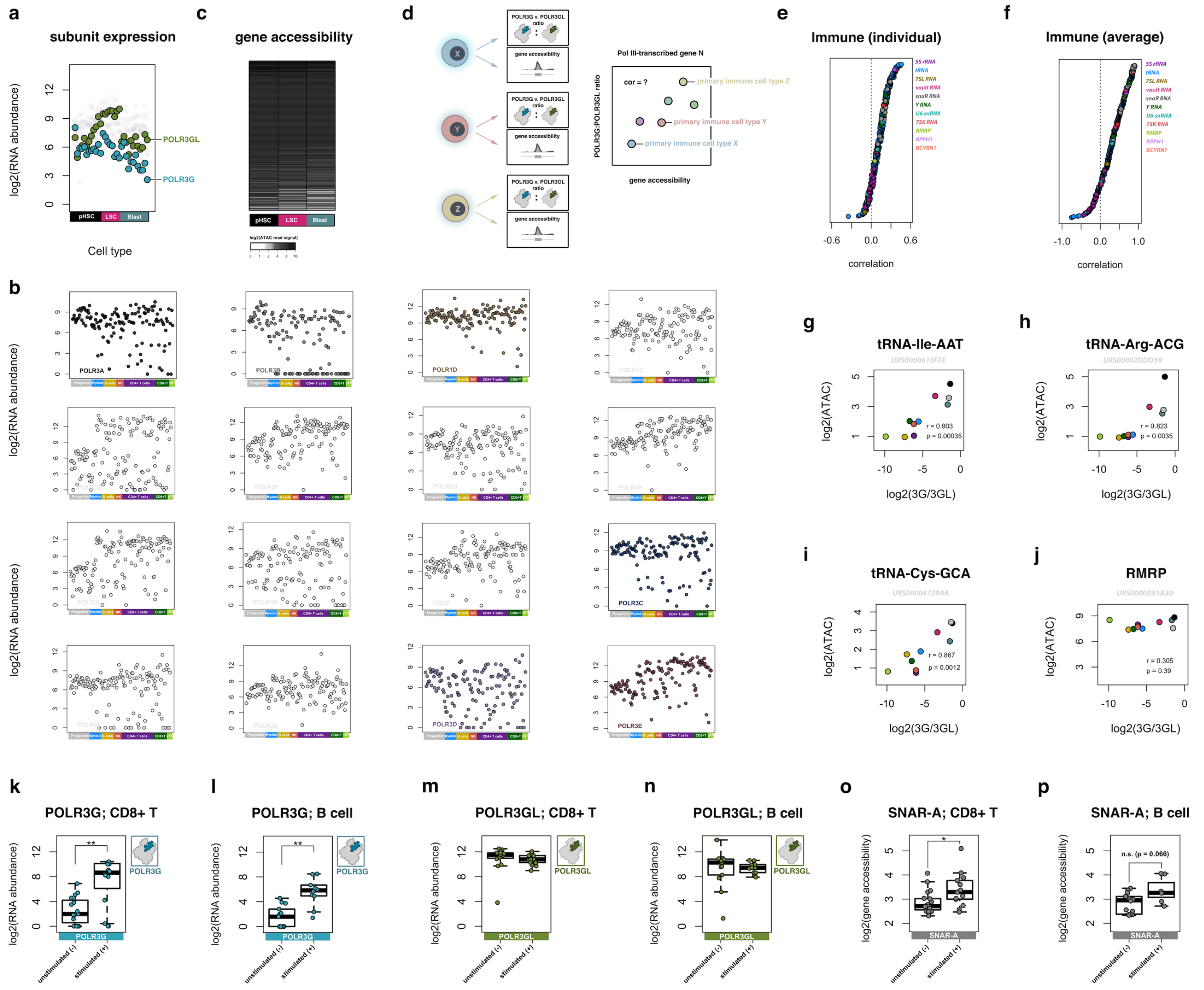

Supplementary Figure 3 I Integrated analysis of Pol III identity and transcriptome chromatin features during primary immune differentiation (**a**) Gene expression levels of mapped Pol III subunits across pre-leukemic hematopoietic stem cells (pHSC), leukemia stem cells (LSC), and Leukemia blast cells (Blast) with emphasis on *POLR3G* and *POLR3GL*. (**b**) Gene expression levels of all other Pol III subunits across primary immune lineages, related to Figure 3A; mapped subunits are colored. (**c**) Gene accessibility measured by ATAC-seq read signal mapping to the repertoire of Pol III occupied genes corresponding to gene expression profiles and cell types in Extended Data Fig. 3a. (**d**) Illustration of approach for integrated analysis of Pol III identity and gene repertoire activity in primary immune cells. *POLR3G* and *POLR3GL* gene expression ratios and ATAC-seq accessibility signals are matched to individual samples and the relationship between these features are assessed by correlation analysis for each individual Pol III-transcribed gene across all profiles samples. (**e**) Resulting correlation scores for Pol III-transcribed genes across all individual samples. (**f**) Resulting correlation scores for Pol III-transcribed genes across aggregate cell-type populations. (**g-j**) Example profiles of individual gene accessibility correlation scores with *POLR3G:POLR3GL* expression ratios in primary immune cells, including (**f**) *tRNA-Ile-AAT* (URS0000610FFE) (**g**) *tRNA-Arg-ACG* (URS00002DDD59) (**h**) *tRNA-Cys-GCA* (URS00004728AA) and (**j**) *RMRP*. Pearson's correlation, corresponding *p*-values computed as two-sided test. (**k-l**) *POLR3G* gene expression in primary CD8+ T cells and primary B cells before and after stimulation (anti-CD3/CD28 and IL-4 respectively). From left to right, N=15,16 (CD8+T) and N=13,10 (B cells) independent experiments. Statistical analysis with a two-sided Wilcoxon rank-sum test. P =

0.0014 (**k**) and 0.00081 (**l**) (**m-n**) *POLR3GL* gene expression in primary CD8+ T cells and primary B cells before and after stimulation. From left to right, N=15,16 (CD8+T) and N=13,10 (B cells) independent experiments. Statistical analysis with a two-sided Wilcoxon rank-sum test. P = 0.059 (**m**) and 0.37 (**n**) (**o-p**) *SNAR-A* gene accessibility profiles measured by ATAC-seq in primary CD8+ T cells and primary B cells before and after stimulation. From left to right, N=19,14 (CD8+T) and N=14,7 (B cells) independent experiments. Statistical analysis with a two-sided Wilcoxon rank-sum test. P = 0.015 (**o**) and 0.066 (**p**). Box plot center lines correspond to median; lower and upper hinges first and third quartile. Whiskers present minimum and maximum values not exceeding 1.5*IQR beyond first and third quartile. *, P ≤ 0.05; **, P ≤ 0.01; ***, P ≤ 0.001; ns, nonsignificant. Source Data are provided as a Source Data file.

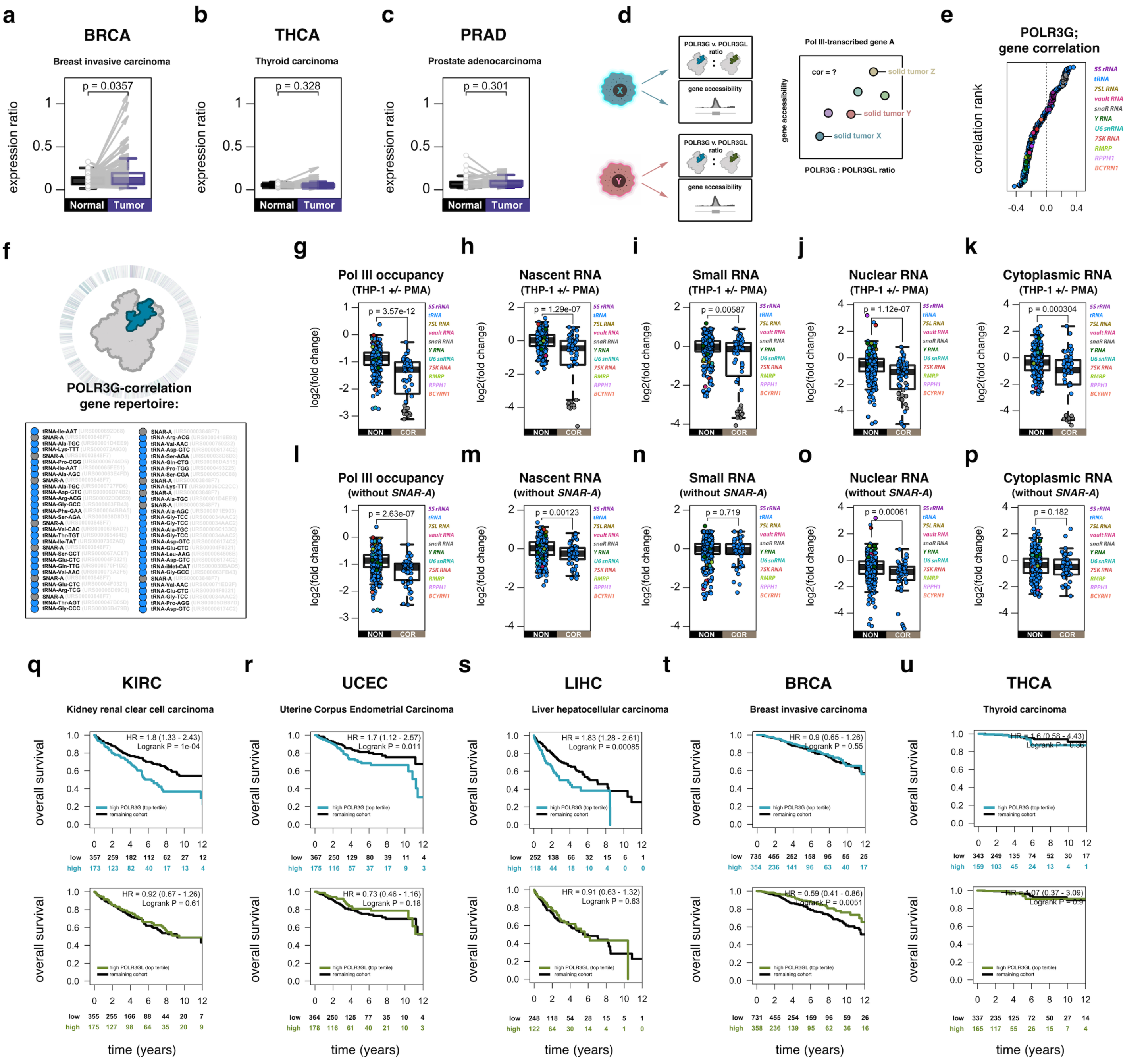

Supplementary Figure 4 l POLR3G expression and correlative chromatin signatures at POLR3G-sensitive gene repertoires in human primary solid tumors (**a-c**) Gene expression ratios of POLR3G/POLR3GL in matched normal tissue and primary solid tumors in breast invasive carcinoma (**a**, n=115), thyroid carcinoma (**b**, n=59), and prostate adenocarcinoma (**c**, n=52) primary solid tumors. Gray arrows represent individual patient-matched normal and primary solid tumors. Corresponding p-values determined using paired Wilcoxon signed-rank two-sided test. Box plot center lines correspond to median; lower and upper hinges first and third quartile. Whiskers present minimum and maximum values not exceeding 1.5*IQR beyond first and third quartile. (**d**) Illustration of approach for integrated analysis of Pol III identity and gene repertoire activity in TCGA data. *POLR3G* and *POLR3GL* gene expression ratios and ATAC-seq accessibility signals are matched to individual samples and the relationship between these features are assessed by correlation analysis. (**e**) Ranked correlation scores for Pol III-transcribed gene accessibility and *POLR3G/POLR3GL* ratios across individual primary solid tumors. Points represent individual Pol III-transcribed genes; gene type indicated by color legend. (**f**) Putative POLR3G-enhanced gene repertoire related to Figure 4k. (**g-p**) Comparison analysis of changes in correlative (COR) and noncorrelative (NON) subgroups (with or without pre-removal of *SNAR-A* genes) related to Pol III occupancy (**g,l**), nascent RNA (**h,m**), steady-state RNA (**i,n**), nuclear (**j,o**), and cytoplasmic RNA (**k,p**) small RNA in THP-1 cells +/- 72h PMA-induced differentiation. Statistical analyses with two-sided Wilcoxon rank-sum tests. N = 290 noncorrelative (NON) genes and 60 correlative (COR) genes including SNAR-A (**g**-**k**), and 48 correlative (COR) genes masking SNAR-A (**l**-**p**). Average log2(fold change) values derived from two biological replicates. Box plot center lines correspond to median; lower and upper hinges first and third quartile. Whiskers present minimum and maximum values not exceeding 1.5*IQR beyond first and third quartile. (**q-u**) Kaplan-Meier analysis of overall survival of TCGA donors stratified by high *POLR3G* expression (top tertile, top) or high *POLR3GL* expression (top terile, bottom) in kidney renal clear cell carcinoma (**q**), uterine corpus endometrial carcinoma (**r**), liver hepatocellular carcinoma (**s**), breast invasive carcinoma (**t**), and thyroid carcinoma (**u**). Corresponding p-values determined using log-rank two-sided test; HR = hazard ratio risk of dying. Source Data are provided as a Source Data file.

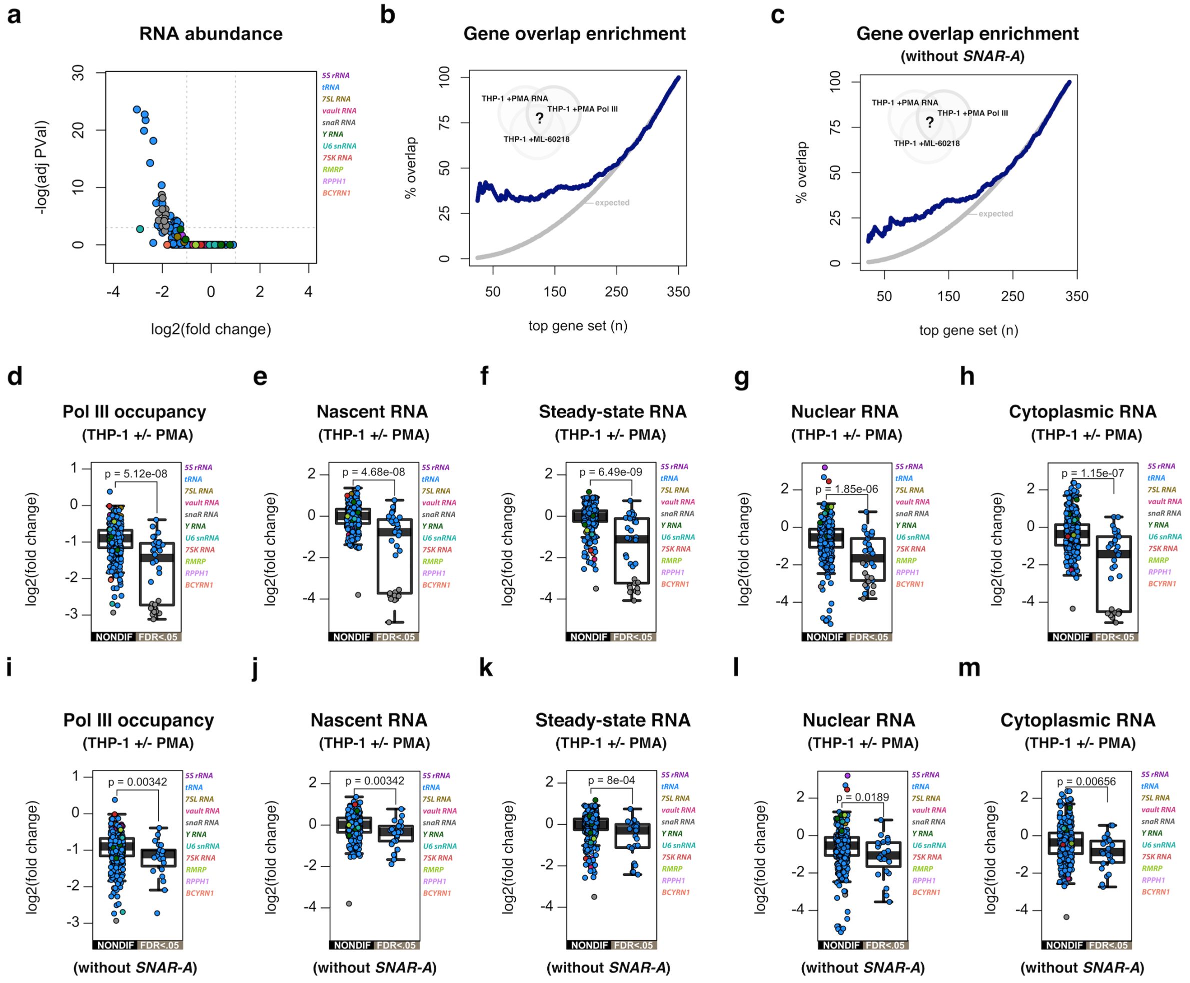

Supplementary Figure 5 I POLR3G disruption mirrors dynamic RNA signatures observed during cell differentiation. (**a**) Volcano plot visualization of small RNA abundance in THP-1 cells +/- 4 h exposure to Pol III inhibitor ML-60218 (25 uM). Significance calculated using edgeR two-sided exact test function, Benjamini-Hochberg corrected p-value. (**b**) Moving Venn-diagram overlap analysis of the dynamic Pol III occupancy and small RNA response observed during THP-1 differentiation (72-hour PMA exposure) with POLR3G disruption effects on THP-1 small RNA levels (4 h ML-60218 exposure). (**c**) Analogous overlap enrichment analysis of the dynamic Pol III occupancy and small RNA response observed during THP-1 differentiation (72-hour PMA exposure) with POLR3G disruption effects on THP-1 small RNA levels (4 h ML-60218 exposure) excluding *SNAR-A* gene representation. (**d-h**) Comparison of the log2(fold change) in Pol III occupancy (**d**), nascent RNA levels (**e**), total steady-state small RNA levels (**f**), nuclear RNA levels (**g**), and cytoplasmic RNA levels (**h**) for nondifferential (NONDIF) and significantly differential (FDR<.05) genes that are sensitive to 4-hour ML-60218 exposure in THP-1 cells, before and after PMA-induced differentiation. (**i-m**) Analogous comparison groups excluding *SNAR-A* genes (related to panels **d-h**). Statistical analyses with two-sided Wilcoxon rank-sum tests. N = 316 nondifferential (NONDIF) genes and 34 differential (FDR<.05) genes including SNAR-A (**d-h**), and 23 differential (FDR<.05) genes masking SNAR-A (**i-m**). Average log2(fold change) values derived from two biological replicates. Box plot center lines correspond to median; lower and upper hinges first and third quartile. Whiskers present minimum and maximum values not exceeding 1.5*IQR beyond first and third quartile. Source Data are provided as a Source Data file.

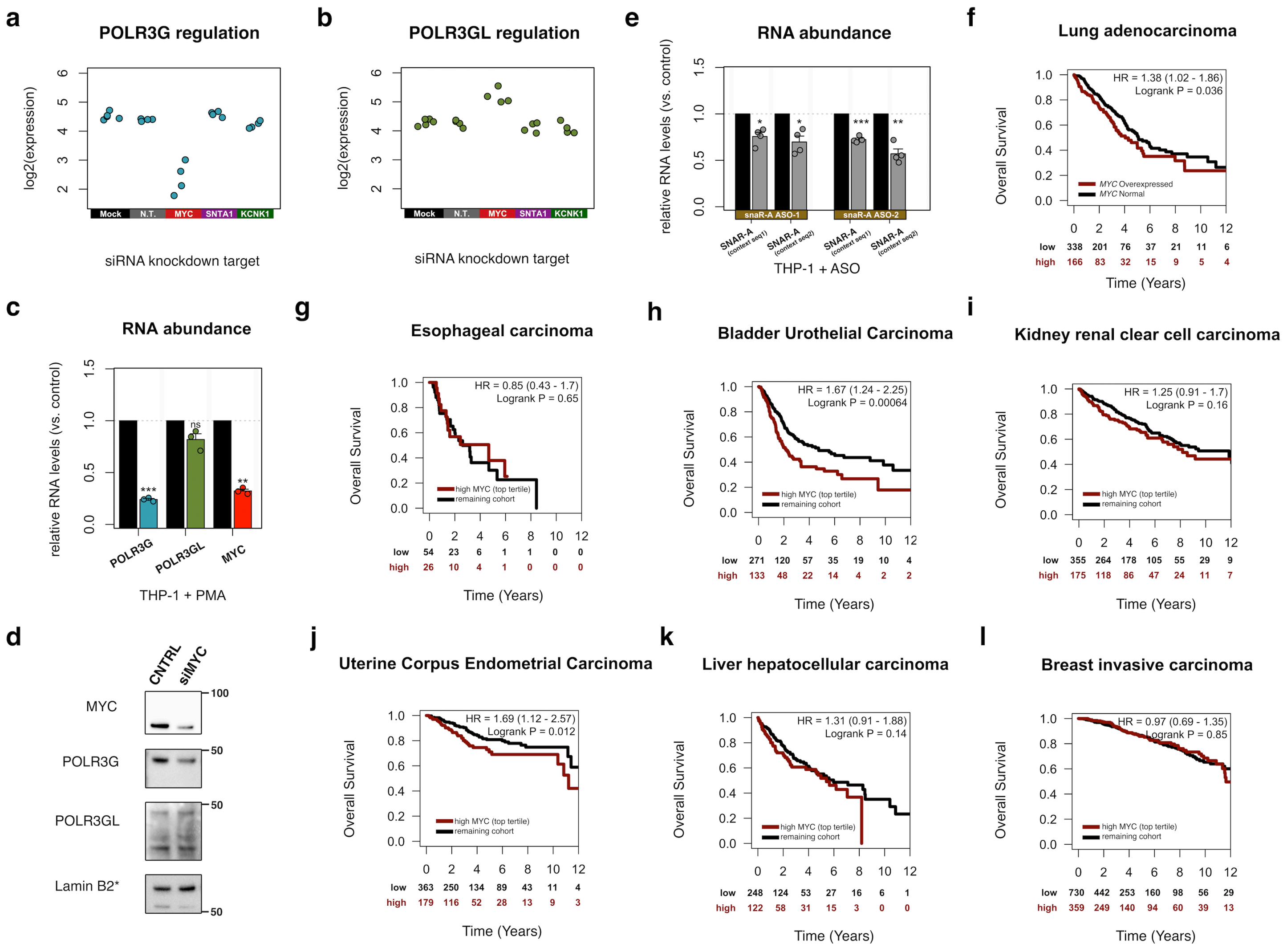

Supplementary Figure 6 l POLR3G levels are associated with poor survival outcomes in specific cancers. (**a-b**) Analysis of MYC and other factor-specific knockdown effects on *POLR3G* (**a**) and *POLR3GL* (**b**) gene expression levels in RKO cells (Topham et al., 2015). Points represent individual biological replicates; N.T. = non-targeting. (**c**) RT-qPCR analysis of *POLR3G*, *POLR3GL*, and *MYC* mRNA levels following THP-1 differentiation (n = 3 biologically independent experiments). P-value = 0.00088 (*POLR3G*), 0.099 (*POLR3GL*), 0.0021 (*MYC*). Significance calculated using Student two-sided t test. (**d**) Immunoblot for Pol III subunits POLR3G and POLR3GL in THP-1 cells, +/- siMYC experiments. *Lamin B2 loading control corresponds to POLR3G. Observations representative of 3 independent experiments (**e**) RT-qPCR analysis snaR-A ncRNA levels in THP-1 cells transfected with ASOs targeting conserved sequences within the core (ASO-1) and tail (ASO-2) regions of snaR-A ncRNA, from left-to-right P = 0.019, 0.027, 0.00067, 0.0071. Significance calculated using Student two-sided t test. Data are presented as mean +/- standard error mean (SEM) from the indicated number of independent samples (**c** and **e**). *, P ≤ 0.05; **, P ≤ 0.01; ***, P ≤ 0.001; ns, nonsignificant. (**f-l**) Kaplan-Meier analysis of overall survival of TCGA donors stratified by high *MYC* expression (top tertile) and normal *MYC* expression in lung adenocarcinoma (**f**), esophageal carcinoma (**g**), bladder urothelial carcinoma (**h**), kidney renal clear cell carcinoma (**i**), uterine corpus endometrial carcinoma (**j**), liver hepatocellular carcinoma (**k**), and breast invasive carcinoma (**l**). Corresponding p-values determined using log-rank two-sided test; HR = hazard ratio risk of dying. Molecular weights are indicated in kDa. *, P ≤ 0.05; **, P ≤ 0.01; ***, P ≤ 0.001; ns, nonsignificant. Source Data are provided as a Source Data file.
