# Supplementary material for: A cancer-associated RNA polymerase III identity drives robust transcription and expression of snaR-A noncoding RNA
Source: Nat Commun. 2022 May 30;13:3007. doi: 10.1038/s41467-022-30323-6 (PMC9151912; doi:10.1038/s41467-022-30323-6)
Supplement: Supplementary file 2 — Reporting Summary [file 41467_2022_30323_MOESM2_ESM.pdf]

nature portfolio

Corresponding author(s): Michael Snyder

Last updated by author(s): Mar 20, 2022

Reporting Summary

Nature Portfolio wishes to improve the reproducibility of the work that we publish. This form provides structure for consistency and transparency in reporting. For further information on Nature Portfolio policies, see our Editorial Policies and the Editorial Policy Checklist.

Statistics

For all statistical analyses, confirm that the following items are present in the figure legend, table legend, main text, or Methods section.

n/a	Confirmed	
☐	☒	The exact sample size (n) for each experimental group/condition, given as a discrete number and unit of measurement
☐	☒	A statement on whether measurements were taken from distinct samples or whether the same sample was measured repeatedly
☐	☒	The statistical test(s) used AND whether they are one- or two-sided<br>*Only common tests should be described solely by name; describe more complex techniques in the Methods section.*
☒	☐	A description of all covariates tested
☐	☒	A description of any assumptions or corrections, such as tests of normality and adjustment for multiple comparisons
☐	☒	A full description of the statistical parameters including central tendency (e.g. means) or other basic estimates (e.g. regression coefficient) AND variation (e.g. standard deviation) or associated estimates of uncertainty (e.g. confidence intervals)
☐	☒	For null hypothesis testing, the test statistic (e.g. F, t, r) with confidence intervals, effect sizes, degrees of freedom and P value noted<br>*Give P values as exact values whenever suitable.*
☒	☐	For Bayesian analysis, information on the choice of priors and Markov chain Monte Carlo settings
☒	☐	For hierarchical and complex designs, identification of the appropriate level for tests and full reporting of outcomes
☐	☒	Estimates of effect sizes (e.g. Cohen's d, Pearson's r), indicating how they were calculated

Our web collection on statistics for biologists contains articles on many of the points above.

Software and code

Policy information about availability of computer code

Data collection	Real-time Quantitative PCR was performed with a QuantStudio 6 Flex Real-Time PCR System (Applied Biosystems)
Data analysis	Genomic analyses were performed using open source software:<br>- trimgalore v0.4.0<br>- Bowtie v2.2.4<br>- samtools v1.3.1<br>- bedtools v2.23.0<br>- ucsc_tools v3.0.9<br>- deepTools v2.2<br><br>R software and packages: R v 4.1.2, edgeR v 3.36.0, Sushi v 1.31.0, surviplot v 1.1.1, plot3D v 1.4, survival 3.2-13, gplots 3.1.1

For manuscripts utilizing custom algorithms or software that are central to the research but not yet described in published literature, software must be made available to editors and reviewers. We strongly encourage code deposition in a community repository (e.g. GitHub). See the Nature Portfolio guidelines for submitting code & software for further information.

Data

Policy information about availability of data

All manuscripts must include a data availability statement. This statement should provide the following information, where applicable:

- Accession codes, unique identifiers, or web links for publicly available datasets
- A description of any restrictions on data availability
- For clinical datasets or third party data, please ensure that the statement adheres to our policy

All data generated for this study are publicly available in the Gene Expression Omnibus under accession codes GSE163422 (Pol III ChIP-seq) and GSE171884 (small RNA-seq)

Previously mapped genomic features in THP-1
- Gene Expression Omnibus series GSE96800

Human hg38 ncRNA gene annotations
- RNAcentral database release version 13 (ftp://ftp.ebi.ac.uk/pub/databases/RNAcentral/releases/13.0/genome_coordinates/bed/homo_sapiens.GRCh38.bed.gz)

Gene expression and ATAC-seq data in primary immune progenitor and differentiated cell populations
- Gene Expression Omnibus series GSE74912 and GSE118119

NIH Roadmap Epigenomics multi-tissue gene expression
- https://egg2.wustl.edu/roadmap/web_portal/ /processed_data.html#RNAseq_uni_proc (file 57epigenomes.RPKM.pc)

The Cancer Genome Atlas (TCGA) gene expression
- Broad Institute TCGA Genome Data Analysis Center (GDAC) Firehose mRNASeq Level 3 RSEM gene normalized data files (https://gdac.broadinstitute.org)

The Cancer Genome Atlas (TCGA) ATAC-seq
Genomic Data Commons Data Portal (https://portal.gdc.cancer.gov)

The Cancer Genome Atlas (TCGA) survival outcome data
- Kaplan-Meier Plotter Pan-cancer RNA-seq analysis platform (https://kmplot.com/analysis/index.php?p=service&cancer=pancancer_rnaseq)

Field-specific reporting

Please select the one below that is the best fit for your research. If you are not sure, read the appropriate sections before making your selection.

☒ Life sciences ☐ Behavioural & social sciences ☐ Ecological, evolutionary & environmental sciences

For a reference copy of the document with all sections, see nature.com/documents/nr-reporting-summary-flat.pdf

Life sciences study design

All studies must disclose on these points even when the disclosure is negative.

Sample size	Experiments relying on and reporting statistical significance were performed in biological duplicate, triplicate, or greater (where noted) to assess statistical significance, relying on protocols established in previous publications, the amount of starting material required, and generally-accepted approaches.
Data exclusions	No data was excluded from analysis
Replication	Experiments performed in this manuscript were repeated independently with 2 or more biological replicates, all attempts at replication were successful. For genomic studies, deposited data also include sequencing replicates to increase coverage for specific experiments, sequencing replicates were combined prior to comparison across biological replicates.
Randomization	Sample allocation was random with respect to treatment condition, control and treatment group experiments were performed in tandem and subsequently analyzed together
Blinding	Blinding was not performed due to use of non-subjective means of quantification and experiments on cell cultures without human /animal subjectivity

Reporting for specific materials, systems and methods

We require information from authors about some types of materials, experimental systems and methods used in many studies. Here, indicate whether each material, system or method listed is relevant to your study. If you are not sure if a list item applies to your research, read the appropriate section before selecting a response.

Materials & experimental systems

n/a	Involved in the study
☐	☒ Antibodies
☐	☒ Eukaryotic cell lines
☒	☐ Palaeontology and archaeology
☒	☐ Animals and other organisms
☒	☐ Human research participants
☒	☐ Clinical data
☒	☐ Dual use research of concern

Methods

n/a	Involved in the study
☐	☒ ChIP-seq
☒	☐ Flow cytometry
☒	☐ MRI-based neuroimaging

Antibodies

Antibodies used

ChIP-seq (5 ug antibody per ChIP experiment/replicate:)
IgG (NeoMarkers NC-100-P0)
BRF1 (Abcam, ab74221)
GTFC3 (SigmaAldrich PLA0180)
POLR3A (Abcam, ab96328 lot#GR318563)
POLR3B (Bethyl, A301-855A)
POLR1D (Bethyl, A304-847A)
POLR3C (Bethyl, A303-063A)
POLR3D (Bethyl, A302-295A)
POLR3E (Bethyl, A303-708A)
POLR3G (Invitrogen, PA5-51120 lot#UG2803044)
POLR3GL (Novus Biologicals, NBP1-79826)

Immunostaining:
Rabbit Anti-POLR3A (Abcam, ab96328 lot#GR318563). Used at 1:1,000
Rabbit Anti-POLR3B (Bethyl, A301-855A). Used at 1:1,000
Rabbit Anti-POLR3C (Bethyl, A303-063A). Used at 1:1,000
Rabbit Anti-POLR3D (Bethyl, A302-295A). Used at 1:1,000
Rabbit Anti-POLR3E (Bethyl, A303-708A). Used at 1:333
Rabbit Anti-POLR3G (Invitrogen, PA5-51120 lot#UG2803044). Used at 1:2,500
Rabbit Anti-POLR3GL (Novus Biologicals, NBP1-79826). Used at 1:150
Rabbit Anti-MYC (Novus Biologicals, NBP2-43691). Used at 1:1,000
Rabbit Anti-LaminB2 (Cell Signaling Technologies, E1S1Q lot 1). Used at 1:1,000
Rabbit Anti-TUBB (Abcam, ab21058, lot# GR3280069-1). Used at 1:5,000

Validation

Anti-POLR3A (Abcam, ab96328 lot#GR318563) amino acids 182 and 452 of POLR3A, affinity purified and validated by manufacturer by immunoblot using human samples, and validated in previous publications (Nat Commun 11:6409)
Anti-POLR3B (Bethyl, A301-855A) between amino acids 1083 and 1133, affinity purified and validated by manufacturer by IP, immunohistochemistry, and immunoblot using human samples, (no citations listed)
Anti-POLR1D (Bethyl, A304-847A) between amino acids 1 and 50, affinity purified and validated by manufacturer by immunoblot using human samples, (no citations listed)
Anti-POLR3C (Bethyl, A303-063A) between amino acids 484 and 534, affinity purified and validated by manufacturer by IP and immunoblot using human samples, (no citations listed)
Anti-POLR3D (Bethyl, A302-295A) between amino acids 175 and 225, affinity purified and validated by manufacturer by IP, immunohistochemistry, and immunoblot using human samples, (no citations listed)
Anti-POLR3E (Bethyl, A303-708A) between amino acids 475 and 525, affinity purified and validated by manufacturer by immunoblot using human samples, (no citations listed)
Anti-POLR3G (Invitrogen, PA5-51120 lot#UG2803044) Synthesized peptide derived from the Internal region of human POLR3G; affinity purified and validated by manufacturer by immunoblot on human samples, (no citations listed). Our study confirms coinciding loss of POLR3G mRNA and protein levels (immunostaining, this antibody) in THP-1 cells
Anti-POLR3GL (Novus Biologicals, NBP1-79826) Peptide sequence DEEKEEEEEKEEEEEEEYDEEEHEEETDYIMSYFDNGEDFGGDSDDNMDE; affinity purified and validated by manufacturer by immunoblot on human samples, (no citations listed). Our study confirms consistent protein levels following depletion of paralogous subunit POLR3G, confirming specificity in THP-1 cells.
Anti-MYC (Novus Biologicals, NBP2-43691) Synthetic peptide corresponding to a region within amino acids 410 and 419 of human c-Myc, affinity purified and validated by manufacturer by immunoblot on human samples, (no citations listed). Our study confirms coinciding loss of MYC protein levels (immunostaining, this antibody) following MYC KD
Anti-LaminB2 (Cell Signaling Technologies, E1S1Q lot 1) synthetic peptide corresponding to residues surrounding Leu75 of human lamin B2 protein, validated by manufacturer by immunoblot using human samples and in previous publications (Sci Signal 11;14(682))
Anti-TUBB (Abcam, ab21058, lot# GR3280069-1) Synthetic peptide corresponding to Human beta Tubulin aa 1-100 conjugated to

keyhole limpet haemocyanin, validated by manufacturer by immunoblot using mouse and human samples, and previously validated in several publications (Dev Cell 56:1727-1741.e7)

Eukaryotic cell lines

Policy information about cell lines

Cell line source(s)	THP-1 was obtained from ATCC (lot # 62454382)
Authentication	THP-1 cells (TIB-202, Lot# 62454382) were authenticated by ATCC, including STR analysis, COI analysis, and morphology
Mycoplasma contamination	THP-1 cells were confirmed to be mycoplasma negative prior to experiments
Commonly misidentified lines (See ICLAC register)	No cell lines registered on ICLAC were used in this study

ChIP-seq

Data deposition

☒ Confirm that both raw and final processed data have been deposited in a public database such as GEO.

☒ Confirm that you have deposited or provided access to graph files (e.g. BED files) for the called peaks.

Data access links
May remain private before publication.

https://www.ncbi.nlm.nih.gov/geo/query/acc.cgi?acc=GSE171884
https://www.ncbi.nlm.nih.gov/geo/query/acc.cgi?acc=GSE163422

Files in database submission

GSM5236051 Total_SmallRNA_THP1_monocyte_1
GSM5236052 Total_SmallRNA_THP1_monocyte_2
GSM5236053 Total_SmallRNA_THP1_macrophage_1
GSM5236054 Total_SmallRNA_THP1_macrophage_2
GSM5236055 Nuclear_SmallRNA_THP1_monocyte_0h
GSM5236056 Nuclear_SmallRNA_THP1_monocyte_1h
GSM5236057 Nuclear_SmallRNA_THP1_monocyte_4h
GSM5236058 Nuclear_SmallRNA_THP1_monocyte_24h
GSM5236059 Nuclear_SmallRNA_THP1_macrophage_72h
GSM5236060 Cytoplasm_SmallRNA_THP1_monocyte_0h
GSM5236061 Cytoplasm_SmallRNA_THP1_monocyte_1h
GSM5236062 Cytoplasm_SmallRNA_THP1_monocyte_4h
GSM5236063 Cytoplasm_SmallRNA_THP1_monocyte_24h
GSM5236064 Cytoplasm_SmallRNA_THP1_macrophage_72h
GSM5236065 Exosomal_SmallRNA_THP1_monocyte_1_1
GSM5236066 Exosomal_SmallRNA_THP1_monocyte_1_2
GSM5236067 Exosomal_SmallRNA_THP1_monocyte_1_3
GSM5236068 Exosomal_SmallRNA_THP1_monocyte_2_1
GSM5236069 Exosomal_SmallRNA_THP1_monocyte_2_2
GSM5236070 Exosomal_SmallRNA_THP1_monocyte_2_3
GSM5236071 Exosomal_SmallRNA_THP1_monocyte_3
GSM5236072 Exosomal_SmallRNA_THP1_macrophage_1_1
GSM5236073 Exosomal_SmallRNA_THP1_macrophage_1_2
GSM5236074 Exosomal_SmallRNA_THP1_macrophage_1_3
GSM5236075 Exosomal_SmallRNA_THP1_macrophage_2_1
GSM5236076 Exosomal_SmallRNA_THP1_macrophage_2_2
GSM5236077 Exosomal_SmallRNA_THP1_macrophage_2_3
GSM5236078 Exosomal_SmallRNA_THP1_macrophage_3
GSM5236079 Total_SmallRNA_THP1_ML602178_0h_1
GSM5236080 Total_SmallRNA_THP1_ML602178_0h_2
GSM5236081 Total_SmallRNA_THP1_ML602178_1h_1
GSM5236082 Total_SmallRNA_THP1_ML602178_1h_2_1
GSM5236083 Total_SmallRNA_THP1_ML602178_1h_2_2
GSM5236084 Total_SmallRNA_THP1_ML602178_2h_1
GSM5236085 Total_SmallRNA_THP1_ML602178_2h_2_1
GSM5236086 Total_SmallRNA_THP1_ML602178_2h_2_2
GSM5236087 Total_SmallRNA_THP1_ML602178_3h_1
GSM5236088 Total_SmallRNA_THP1_ML602178_3h_2_1
GSM5236089 Total_SmallRNA_THP1_ML602178_3h_2_2
GSM5236090 Total_SmallRNA_THP1_ML602178_4h_1
GSM5236091 Total_SmallRNA_THP1_ML602178_4h_2_1
GSM5236092 Total_SmallRNA_THP1_ML602178_4h_2_2
GSM4979114 POLR3A_monocyte_1
GSM4979115 POLR3A_monocyte_2
GSM4979116 POLR3B_monocyte_1
GSM4979117 POLR3B_monocyte_2
GSM4979118 POLR1D_monocyte_1

GSM4979119 POLR1D_monocyte_2
GSM4979120 POLR3C_monocyte_1
GSM4979121 POLR3C_monocyte_2
GSM4979122 POLR3G_monocyte_1
GSM4979123 POLR3G_monocyte_2
GSM4979124 POLR3GL_monocyte_1
GSM4979125 POLR3GL_monocyte_2
GSM4979126 POLR3D_monocyte_1
GSM4979127 POLR3D_monocyte_2
GSM4979128 POLR3E_monocyte_1
GSM4979129 POLR3E_monocyte_2
GSM4979130 BRF1_monocyte
GSM4979131 GTF3C1_monocyte
GSM4979132 IgG_monocyte
GSM4979133 POLR3B_macrophage_1
GSM4979134 POLR3B_macrophage_2
GSM4979135 POLR1D_macrophage_1
GSM4979136 POLR1D_macrophage_2
GSM4979137 POLR3G_macrophage_1
GSM4979138 POLR3G_macrophage_2
GSM4979139 POLR3GL_macrophage_1
GSM4979140 POLR3GL_macrophage_2
GSM4979141 POLR3D_macrophage_1
GSM4979142 POLR3D_macrophage_2
GSM4979143 POLR3G_monocyte_ML60218_1
GSM4979144 POLR3G_monocyte_ML60218_2
GSM4979145 POLR3GL_monocyte_ML60218_1
GSM4979146 POLR3GL_monocyte_ML60218_2

Genome browser session
(e.g. UCSC)

All data are publicly available, we did not use UCSC sessions

Methodology

Replicates

Each ChIP-seq experiment, with the exception of BRF1 and GTF3C1 (where no statistical or dynamic binding features were inferred), were performed in duplicate.

Sequencing depth

GSM4979114 POLR3A_monocyte_1: 42,543,872 - 101bp, PE, Sequenced a second time: 22,569,749 - 101bp, PE
GSM4979115 POLR3A_monocyte_2: 58,150,077 - 101bp, PE, Sequenced a second time: 27,170,844 - 101bp, PE
GSM4979116 POLR3B_monocyte_1: 75,863,661 - 101bp, PE
GSM4979117 POLR3B_monocyte_2: 88,376,122 - 101bp, PE
GSM4979118 POLR1D_monocyte_1: 79,837,738 - 101bp, PE
GSM4979119 POLR1D_monocyte_2: 80,828,932 - 101bp, PE
GSM4979120 POLR3C_monocyte_1: 73,275,866 - 101bp, PE
GSM4979121 POLR3C_monocyte_2: 86,527,794 - 101bp, PE
GSM4979122 POLR3G_monocyte_1: 37,084,297 - 101bp, PE
GSM4979123 POLR3G_monocyte_2: 49,872,619 - 101bp, PE
GSM4979124 POLR3GL_monocyte_1: 49,548,081 - 101bp, PE
GSM4979125 POLR3GL_monocyte_2: 52,538,798 - 101bp, PE
GSM4979126 POLR3D_monocyte_1: 68,454,694 - 101bp, PE
GSM4979127 POLR3D_monocyte_2: 88,917,062 - 101bp, PE
GSM4979128 POLR3E_monocyte_1: 69,229,775 - 101bp, PE
GSM4979129 POLR3E_monocyte_2: 78,797,903 - 101bp, PE
GSM4979130 BRF1_monocyte: 88,311,209 - 101bp, PE
GSM4979131 GTF3C1_monocyte: 125,973,940 - 101bp, PE
GSM4979132 IgG_monocyte: 42,381,774 - 101bp, PE
GSM4979133 POLR3B_macrophage_1: 76,712,848 - 101bp, PE
GSM4979134 POLR3B_macrophage_2: 82,759,430 - 101bp, PE
GSM4979135 POLR1D_macrophage_1: 75,403,048 - 101bp, PE
GSM4979136 POLR1D_macrophage_2: 75,489,289 - 101bp, PE
GSM4979137 POLR3G_macrophage_1: 36,383,080 - 101bp, PE
GSM4979138 POLR3G_macrophage_2: 40,888,812 - 101bp, PE
GSM4979139 POLR3GL_macrophage_1: 42,876,338 - 101bp, PE
GSM4979140 POLR3GL_macrophage_2: 47,603,671 - 101bp, PE
GSM4979141 POLR3D_macrophage_1: 83,702,011 - 101bp, PE
GSM4979142 POLR3D_macrophage_2: 78,996,699 - 101bp, PE
GSM4979143 POLR3G_monocyte_ML60218_1: 43,947,325 - 101bp, PE
GSM4979144 POLR3G_monocyte_ML60218_2: 52,494,871 - 101bp, PE
GSM4979145 POLR3GL_monocyte_ML60218_1: 53,356,917 - 101bp, PE
GSM4979146 POLR3GL_monocyte_ML60218_2: 57,226,274 - 101bp, PE

Antibodies

ChIP-seq:
Rabbit Anti-POLR3A (Abcam, ab96328 lot#GR318563)
Rabbit Anti-POLR3B (Bethyl, A301-855A)
Rabbit Anti-POLR3C (Bethyl, A303-063A)

Rabbit Anti-POLR3D (Bethyl, A302-295A)
Rabbit Anti-POLR3E (Bethyl, A303-708A)
Rabbit Anti-POLR3G (Invitrogen, PA5-51120 lot#UG2803044)
Rabbit Anti-POLR3GL (Novus Biologicals, NBP1-79826)
Rabbit Anti-GTF3C1 (Sigma-Aldrich, PLA0180)
Rabbit Anti-BRF1 (Abcam, ab74221)
Rabbit IgG (Thermo Scientific, NeoMarkers NC-100-P0)

Peak calling parameters

We did not call peaks, rather Pol III subunit ChIP-seq profiles were extracted over ncRNA gene coordinates. Corresponding aligned bigwig signal files were deposited on GEO GSE163422

Data quality

Peak-calling was not a feature of our study, quality of ChIP-seq was assessed by appropriate signal enrichment over known target Pol III-transcribed genes

Software

ChIP-seq reads were trimmed using trim galore v0.4.0 and aligned to GRch38 using bowtie v2.2.4 with setting "bowtie2 -t sensitive -x".
